# Preparation, characterization and in-vivo evaluation of nanoemulsion based hydrogel of ferulic acid in imiquimod-induced psoriasis in rats

**DOI:** 10.1038/s41598-026-57435-z

**Published:** 2026-06-10

**Authors:** Nosheen Naseer, Sairah Hafeez Kamran, Misbah Sultana, Saiqa Ishtiaq, Farhan Siddique, Muhammad Farhan Sohail, M. Ismail Mastoi

**Affiliations:** 1 Institute of Pharmacy, Faculty of Pharmaceutical and Allied Health Sciences, Lahore College for Women University, Lahore, 54000 Punjab Pakistan; 2 Punjab University College of Pharmacy, University of the Punjab, Lahore, 54000 Punjab Pakistan; 3 Department of Pharmaceutical Chemistry, Faculty of Pharmacy, Bahauddin Zakariya University, Multan, 60800 Pakistan; 4 Alliant College of Professional Studies, Lahore, Punjab Pakistan; 5 Allmed (Pvt.) Ltd. Sunder Industrial Estate, Raiwind Road, Lahore, Pakistan

**Keywords:** Psoriasis, Ferulic acid, Inflammation, Hydrogel, Nano-emulsion, Biochemistry, Biotechnology, Drug discovery, Immunology, Medical research

## Abstract

Psoriasis is skin inflammation caused by an immune system dysfunction, involving a complex interaction between immune cells and keratinocytes. The aim of this study was to develop nanoemulsion (NE) based hydrogel containing ferulic acid, a poorly water-soluble compound and evaluate its effect on imiquimod induced psoriasis like skin inflammation. Ferulic acid nanoemulsion (FA-NE) was prepared by ultra-sonication method. Castor oil, Tween-80 and propylene glycol were selected for NE preparation as oil phase surfactant and cosurfactant respectively based on solubility. The NE particle size (29.8 ± 0.49 nm) was within colloidal range, polydispersity index (0.21 ± 0.02) showed homogenous NE, zeta potential (28.5 ± 0.57 mV) indicated stable NE. Entrapment efficiency (96.26 ± 0.33%) and pH (6.8 ± 0.15) was also in suitable range. The rheological study results of FA-NE hydrogel indicated pseudoplastic behavior. The pH and spreadibility were 6.2 ± 0.15 and 8.2 ± 0.89 respectively, suitable for topical application. The optimized FA-NE hydrogel was tested on male Wistar rats (100–150 g) divided into five groups (*n* = 5). Psoriasis like disease was induced by applying 5% imiquimod (IMQ) cream for 7 days on the shaved dorsal skin of four groups (except normal group) and psoriasis area severity index (PASI) scoring were calculated. The application of optimized FA-NE hydrogel dramatically reduced TNF-α, IL-6, IL-17 A, IL-23, superoxide dismutase, glutathione peroxidase, malondialdehyde and catalase biomarkers when compared to the disease group. These results were consistent with the in-silico docking studies with highest docking score observed with TNF-α. The treatment group’s spleen index reduced significantly. Histopathological findings showed increased capillaries, skin thinning, small dermal projections, and inflammatory cells around blood vessels with 5% IMQ cream application and FA-NE hydrogel greatly reduced these psoriatic changes. Immunohistochemical Ki-67 analysis demonstrated that disease control rats expressed more protein than normal, treatment, and standard animals. The FA-NE hydrogel effectively reduced inflammation and oxidative stress in a rat model of psoriasis, suggesting its potential as a topical treatment.

**Supplementary Information:**

The online version contains supplementary material available at 10.1038/s41598-026-57435-z.

## Introduction

Psoriasis and inflammation are closely linked, T-cell activation playing a key role in both processes^1^. Approximately 2 to 3% of the global population is affected by this dermatological condition. It detrimentally affects a patient’s emotional, psychological, and physical well-being, among other facets of their health^2^. Psoriasis is mostly attributed to a synergistic interplay of genetic, oxidative stress, immunological, and environmental variables. It is asserted that inflammation related to oxidative stress and inflammation connected to immunology substantially contributes to the development of the illness^3^. Psoriasis is associated with an increased concentration of oxidative agents, such as nitric oxide (NO), malondialdehyde (MDA), and inducible nitric oxide synthase (iNOS) which lead to the production of reactive oxygen species (ROS) and free radical burden. The levels of antioxidant enzymes, such as superoxide dismutase (SOD), catalase (CAT) and glutathione peroxidase (GSH), decrease in psoriatic lesions or serum due to free radical burden. ROS and immune dysregulation are responsible for the activation of T helper (Th)1/Th17 cells, which promote the production of inflammatory cytokines such as IL-6, IL-17, IL-22, IL-23, and tumor necrosis factor- alpha (TNF-α)^4^. The Th17 secreted upregulates IL-17 levels which stimulate the proliferation of keratinocytes and subsequently signals the activation of Th1 and Th22 cells. These contribute to the upregulation of IL-23, Il-17 A and TNF-α signaling. Recent clinical trials have demonstrated the clinical efficacy of drugs targeting these signaling pathways in ameliorating psoriatic illness^5^. At present, there are multiple conventional treatments for psoriasis therapy, such as topical therapy, nonbiological systemic therapy, phototherapy, and biological therapies but safety concerns have been found to be associated with some therapies. Herbal medicine has experienced a resurgence in recent years due to its less side effects compared to pharmaceutical drugs. Synthetic drugs provide symptomatic alleviation; however, they are associated with several side effects that may compromise patient safety and adherence to treatment^6^.

Currently, numerous diseases are being addressed by drug delivery systems utilizing nanosized drug particles to effectively reach target sites. This has been prominently utilized in cancer therapies, and now other conditions, particularly topical diseases, are being targeted using nanodrug delivery methods. Nanotechnology is an emerging technology employed to address skin problems, supplementing the conventional technique of topical medicine delivery. Similarly, nanoemulsions (NEs) are drug delivery systems that have been widely studied for the transdermal delivery of various therapeutic agents. They incorporate permeation enhancers to improve drug absorption through the skin. NEs are thermodynamically stable, isotropically transparent mixtures composed of oil, water, surfactants, and co-surfactants in appropriate proportions. However, their low viscosity making it unsuitable for application on skin, to overcome this issue gelling agent employed to improve formulation viscosity^7^ The skin serves as a natural barrier, and transdermal medication administration offers a promising alternative route that circumvents first-pass metabolism and enhances drug bioavailability. The process entails accurate administration of medications in a particular region of the dermis by the creation of a reservoir^8^.

Ferulic acid (FA) is a phytonutrient which belongs to the group of hydroxycinnamic acid, a class of phenolic compounds and most prevalent phenolic acids in plants^9^. Owing to its predominant antioxidant and anti-inflammatory properties, it possesses considerable therapeutic potential in the management of diabetes, cancer, pulmonary, and cardiovascular diseases^10^. FA exhibits poor systemic bioavailability, primarily due to its low aqueous solubility and limited gastrointestinal absorption. Additionally, it undergoes rapid hepatic metabolism and is quickly eliminated, further reducing its therapeutic efficacy^11^. FA is categorized as a member of Class II in the Biopharmaceutical Classification System (BCS) with low solubility and high permeability^12^. The pharmacological efficacy of FA, with enhanced bioavailability in topical preparations, has been evaluated and firmly proven in literature^13^. An investigation indicated that a FA-NE hydrogel demonstrated superior permeability and offered enhanced protection against ultraviolet A compared to traditional dosing forms^14^. The present research seeks to develop NE based hydrogel of FA and evaluate the pharmacological efficacy in mitigating imiquimod-induced psoriasis-like inflammation.

## Materials and methods

### Materials

The FA (99%) was purchased from Sigma-Aldrich (CAS: 1325-24-6), whereas Castor oil, Propylene glycol, Tween-80, Purified water, Carbopol 940, and Triethanolamine were provided as a gift by Allmed Pharmaceutical (Pvt.) Ltd, located in Lahore, Pakistan. The imiquimod (IMQ) cream 5% w/w (G-Warts 5% Cream) was bought from Life Pharmacy-Dubai. The Eczemus (tacrolimus) 0.03% ointment, which is the standard for the topical therapy, was purchased from Clinix Plus Pharmacy Lahore-Pakistan. ELISA Kits of IL-6 and IL-23 by FineTest, IL-17 A by EZRIL and TNF-α by Reed Biotech Limited were used for analysis. Vortex mixer (Vortex-Genie 2 SKU-T236), Centrifugation Machine (S3024R DLAB, China), UV-VIS spectrophotometer (UV-1800 Shimadzu), Hot Plate Magnetic Stirrer (Dawn Scientific MS-017), Litesizer TM 500 (Anton Paar GmBH, Austria), HPLC (Agilent Technologies), Rheometer (TA Instruments AR1500ex), pH Meter (Mettler Toledo Seven Compact pH/Ion S 220 pH meter) and Isothermal Shaker (T-3 S) were used for analysis.

### Preformulation screening for nanoemulsion development

The solubility of FA was assessed already published method^14^ with small modification. The solubility of FA was determined in different oils (isostearyl isostearate (ISIS), olive oil, and castor oil, surfactants (tween-80 and labrasol), co-surfactants (propylene glycol and glycerin) by adding excess amount of FA in 10mL oils, surfactants and co-surfactants in transparent vials with rubber stoppers and continuously stirred on a vortex mixer (Vortex-Genie 2 SKU-T236) for 10 min and then placed on an isothermal shaker (T-3 S) at 25ºCfor 72 h. to get equilibrium. The equilibrated samples were subjected to centrifugation at 13000 rpm (S3024R DLAB, China) for duration of 5 min. The supernatant was carefully separated using a 0.45 μm membrane filter and drug concentration was determined by HPLC at a wavelength of 321 nm with already validated method^14^. The mobile phase constituted of acetonitrile and deionized water in a proportion of 35:65, 30:70 and 25:75 v/v (1% glacial acetic acid; pH 2.2) which were sonicated for 15 min and degassed prior to use. The flow rate was adjusted to 1 mL/min and run time was 15 min. The temperature was adjusted at 25 ± 0.5 °C and C18 column (250 mm × 4.6 mm, 5 μm; (Supherisorb, Waters, Ireland) fitted with a C18 guard column (10 × 3.0 mm) was used. The injection volume was 20µL and repeated 3 injections from same preparation were injected. The linearity of the analytical method for FA was evaluated using five concentration levels ranging from 50 to 150% of the target concentration, corresponding to 2.50–7.50 mg/mL. A standard stock solution of FA (5 mg/mL) was prepared in methanol and analyzed under optimized chromatographic conditions. The calibration curve was constructed by plotting peak area versus concentration and demonstrated excellent linearity over the studied range, with a correlation coefficient (r²) of 0.9998 and regression equation Y=120000x + 5000 indicating a strong proportional relationship between analyte concentration and detector response. The precision of the developed analytical method for FAwas evaluated by analyzing six replicate samples at the 100% concentration level (5 mg/mL). The average area under the curve (AUC) of the standard for five injections was 605120. The percentage recovery values obtained for the replicate samples ranged from 99.89% to 100.13%, with an average recovery of 100.03%, demonstrating excellent repeatability of the method. The retention time remained consistent at approximately 5.21 min for all injections, indicating successful chromatographic reproducibility. The specificity of the method was assessed by analyzing standard, sample, and placebo preparations at the 100% concentration level. The placebo preparation showed no detectable interference at the retention time of FA, confirming the specificity of the method.

The limit of detection (LOD) for the FA is 3.125% (0.16 mg/ml), with an average retention time of 5.21 min and a recovery percentage of 3.12% (0.16 mg/ml). The limit of quantitation (LOQ) for FA is 3.125% (0.16 mg/ml), while the recovery percentage is 3.3% (0.17 mg/ml). The details of validation method are provided in supplementary file.

### Development of pseudo ternary phase diagram

Pseudo ternary phase diagrams were constructed to identify NE region using aqueous titration method. Castor oil was used as oil phase, tween 80 as surfactant and propylene glycol as co-surfactant. Three different ratios i.e. 1:1, 1:2, and 2:1 of Smix (tween 80 & propylene glycol) were prepared using magnetic stirrer for 30 min to obtain homogeneous mixture. Different weight ratios of castor oil with Smix (1:9, 2:8, 3:7, 4:6, 5:5, 6:4, 7:3, 8:2, and 9:1) were used to develop pseudo ternary diagrams. Each mixture was gradually titrated with distilled water under continuous stirring till transparency and uniformity was observed^14,15^. The pseudo ternary diagrams were developed with the help of Microsoft Excel (version 2013). The placebo NEs were initially prepared without drug to identify NE regions for formulation optimization. Table 1 shows the formulation of a placebo NE using different ratios of oil and Smix (1:1)^16^.

**Table 1:** Composition of nanoemulsion to develop pseudo ternary phase diagram with Smix (1:1).

Formulation	Oil & Smix	Oil		Smix		Water	
		% (V/V)	ml	% (V/V)	ml	% (V/V)	ml
A1	1 : 9	4.0	0.8	36.0	7.2	60	12
A2	2 : 8	9.0	1.8	36.0	7.2	55	11
A3	3 : 7	16.5	3.3	38.5	7.7	45	9
A4	4 : 6	22.0	4.4	33.0	6.6	45	9
A5	5 : 5	22.5	4.5	22.5	4.5	55	11
A6	6 : 4	30.6	6.1	20.4	4.1	49	9.8
A7	7 : 3	34.3	6.9	14.7	2.9	51	10.2
A8	8 : 2	38.4	7.7	9.6	1.9	52	10.4
A9	9 : 1	45.0	9	5.0	1	50	10

All the formulations (A1 to A9) were formulated with Smix ratios 1:1, 1:2 and 2:1.

### Preparation of drug loaded ferulic acid nanoemulsion

**Fig. 1:**
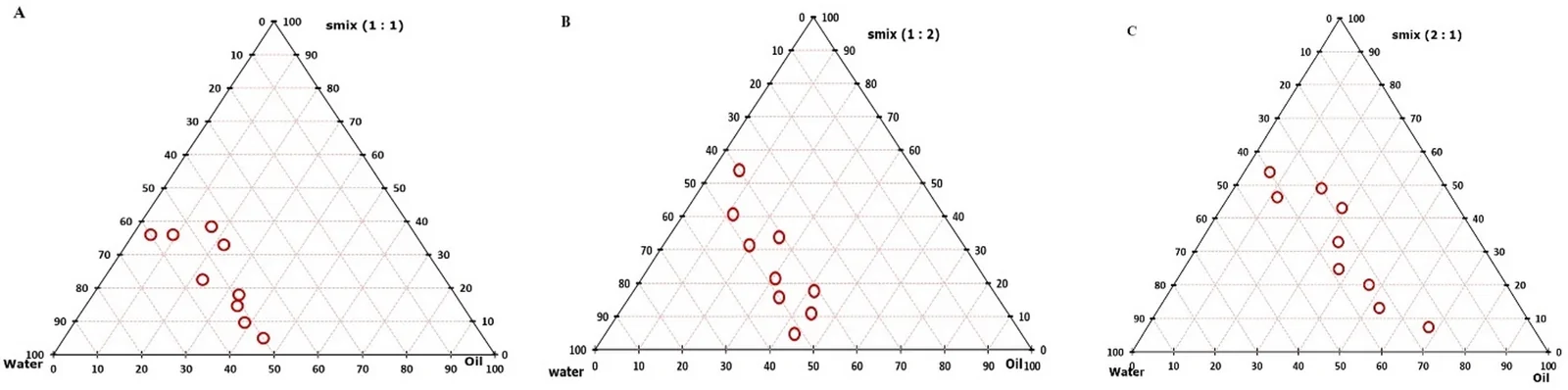
Pseudo-ternary phase diagrams of drug free nanoemulsion prepared with Smix ratio (**A**) 1:1, (**B**) 1:2 & (**C**) 2:1.

With the help of pseudo ternary phase diagram (Fig. 1) the maximum NE regions were found in 5:5 and 6:4 ratio and selected for drug loaded NE formulation. All Smix ratios (1:1, 1:2 and 2:1) were evaluated within these selected regions. The drug loaded NE was prepared by ultra-sonication method^16^ by adding accurate amount of FA (100 mg) in the selected oil. Smix was added in three different ratios (1:1, 1:2 and 2:1) under continuous stirring. The batch size was 20mL and three batches of each formulation (NE1-NE5) were produced under identical manufacturing conditions and methods for characterization. Subsequently, water was added dropwise with continuous agitation until a clear and homogeneous oil-in-water emulsion was achieved. The NEs were afterwards ultrasonicated for an additional two hours. Sonication was performed using an Ultrasonic Cleaner (LAB-O-MED Italiano, Model UC-4120, China), with a frequency of 40 kHz, ultrasonic power of 120 W, heating power of 100 W, and an AC voltage of 220 V at 50/60 Hz. Sonication was conducted in an ice bath to regulate temperature and prevent thermal damage to the formulation. The drug loaded NE formulations are presented in Table 2.

**Table 2:** Composition of ferulic acid loaded nanoemulsion.

Formulation	Oil & Smix Ratio	Oil		Smix		Water		Smix Ratio
		%	ml	%	ml	%	ml	
NE1	5:5	22.5	4.50	22.5	4.50	55	11	2:1
NE2	6:4	30.6	6.1	20.4	4.1	49	9.8	2:1
NE3	5:5	22.5	4.50	22.5	4.50	55	11	1:2
NE4	6:4	30.6	6.1	20.4	4.1	49	9.8	1:2
NE5	5:5	22.5	4.50	22.5	4.50	55	11	1:1
NE6	6:4	30.6	6.1	20.4	4.1	49	9.8	1:1

### Characterization of ferulic acid nanoemulsion

#### Measurement of particle size, polydispersity index and zeta potential

The particle size, polydispersity index (PDI) and zeta potential of the NE1 to NE6 were measured using the Litesizer™ 500 (Anton Paar GmBH, Austria) at 25˚C. All NEs samples were prepared by mixing 1 mL of NE with 9 mL of purified water. The particle size was assessed using dynamic light scattering (DLS) at 25˚C with solvent refractive index of 1.3303 and viscosity 0.00089 This device employs electrophoretic light scattering to ascertain the surface charge of the NE droplets^17,18^.

#### Entrapment efficiency

The entrapment efficiency of FA in the NE was determined by diluting NE with methanol in 10 ml volumetric flask and sonicated the mixture for 15 min at 15 °C^16^. Then centrifuged at 13,000 rpm for 10 min, supernatant analyzed by using high-performance liquid chromatography (HPLC, Agilent Technologies) with a validated method^19^ (Supplementary file). The entrapment efficiency was calculated by using formula^20^;

$${\mathrm{EE}}\;(\% ) = \frac{{{\mathrm{Total}}\;{\mathrm{drug}}\;{\mathrm{initially}}\;{\mathrm{added}}\;({\mathrm{mg}}) - {\mathrm{unentrapped}}\;{\mathrm{drug}}\;({\mathrm{mg}}) \times 100}}{{{\mathrm{Total}}\;{\mathrm{drug}}\;\mathrm{initially} \;{\mathrm{added}}\;({\mathrm{mg}})}}$$

#### Scanning electron microscopy (SEM)

Scanning electron microscopy (SEM) ZEISS (Germany) was determined using^21^ method with slight modification. Hydrogel, NE, and pure drug samples were prepared by placing a small aliquot or fragment onto aluminum SEM stubs and gently air-drying to remove bulk moisture. Solid drug and dried FA-NE hydrogel samples were sputter-coated with Au/Pd (~ 5–10 nm) to prevent charging, while the NE residue was imaged after drying on the stub. Imaging was performed on a ZEISS SEM in high vacuum using appropriate detectors (SE1 for surface morphology, C2DX for compositional contrast) at accelerating voltages up to 20 kV.

#### pH measurement

All NE formulations were tested for pH using a Mettler Toledo Seven Compact pH/Ion S 220 pH meter at room temperature^22^.

#### Phase inversion

The NE phase inversion was tested by dilution method. To evaluate phase inversion, 1 mL of the optimized NE was diluted with 9 mL of purified water^22^.

#### Dispersion stability

The optimized formulations (NE1-NE5) were tested for dispersion stability under heating, cooling, centrifugation (S3024R DLAB, China), and freeze-thaw conditions. For the dispersion stability analysis of NEs, 10 mL of the optimized formulations were held at 4˚C for 48 h and then at 45˚C for the same duration, six times. The mixtures were centrifuged at 3500 rpm for 30 min. The formulations were tested for freeze-thaw stress at temperatures ranging from − 21˚C to + 25˚C for 48 h, three times in Deep Freezer by Qingdao Haier Biomedical Co, Ltd Germany Model No. DW-40L262. After testing, formulations were checked visually for phase separation, cracking, and creaming. The dispersion stability was also analyzed with particle size, PDI, and zeta potential^16^.

### Formulation of nanoemulsion based hydrogel

FA-NE hydrogel was prepared with slight modification^22^. Carbopol-940 was chosen as gelling agent to formulate NE based hydrogel. Based on the particle size and entrapment efficiency, it was determined that all the optimized formulations have nanosize, which makes them suitable for the formulation of hydrogel, however NE5 was utilized for the development of hydrogels, due to good entrapment efficiency and particle size. The Carbopol 940 gel (4%) was initially prepared as stock gel base and allowed to swell for 24 h for uniform hydration of polymer at room temperature.

Five formulations of hydrogels (HG1- HG5) were with constant amount of optimized NE (8 g of NE containing 300 mg of FA), which constituted the specified daily dose (10 mg/Kg) for in-vivo studies. The specified amount of NE was combined with varying proportions of carbopol and water was added to achieve best consistency of hydrogel. The gel was prepared by stirring the mixture at a speed of 500 rpm using silverson mixer (SM-07) until a uniform gel was formed. The pH was adjusted by triethanolamine aqueous solution within a range of 5–6.8. The prepared hydrogels were packed in HDPE bottle with child resistant lid and stored at room temperature. The FA-NE hydrogel composition is provided in (Table 3).

**Table 3:** Composition of Nanoemulsion based hydrogel.

Formulations	NE5		4% Carbopol 940 gel base		Water	
	(%)	(g)	(%)	g	(%)	g
HG1	80	8	5	0.5	15	1.5
HG2	80	8	7.5	0.75	12.5	1.25
HG3	80	8	10	1	10	1
HG4	80	8	12.5	1.25	7.5	0.75
HG5	80	8	15	1.5	5	0.5

NE: Nanoemulsion; HG: Hydrogel.

### Characterization of ferulic acid nanoemulsion-based hydrogel

#### Rheological study

Rheograms of all formulations were recorded at room temperature (25 °C) using Rheometer (TA Instrument Ltd. AR. 1500 ex) with cone and plate geometry. The shear rate was uniformly increased from 0 to 1000 s − 1 over 1 min and then decreased to zero over 1 min at room temperature. The rheograms were taken 24 h after the preparation of nanoemulgels. The yield values (Pa) of all formulations were also noted at the same time. The apparent viscosity (Pa.s) was observed at different shear rates to study the flow characteristics of nanoemulgels^23^.

#### pH determination

The FA-NE hydrogel was intended for topical application, measuring its pH is essential to ensure it is non-irritating to the skin. The pH of the formulation was measured at room temperature using a digital pH meter. The pH meter probe was directly inserted in the gel and the pH value was recorded (Mettler Toledo Seven Compact pH/Ion S 220 pH meter)^21^.

#### Spreadability

To determine the spreadability of all gel formulations, a circle with a diameter of 1 cm was marked on the glass plate. A measured quantity of 500 mg was placed on one plate, and a second plate was placed on top of it, applying pressure for 1 min. The spreadability was determined by increasing the diameter after 1 min, using the following equation.

$${\mathrm{S}} = {\mathrm{m}} \times {\mathrm{D}}/{\mathrm{t}}$$

where m is weight amount of gel (g), D is diameter of the gel (cm) and t (sec) is the holding time^24^.

#### In-vitro skin permeation study

In-vitro skin permeation study was conducted as per earlier method^25^ with slight modification. A Franz diffusion cell (vertical diffusion cell, China) was used for in-vitro skin permeation tests because it accurately predicts drug transport across the skin. The dorsal skin of rodents is often utilized for in vitro skin permeation studies due to its accessibility, cost-effectiveness, and appropriate size for laboratory manipulation. The dorsal area of the Wistar rat was shaved and the skin was excised. The surplus fat tissue and connective structures were carefully excised. Following cleaning with standard saline, the skin was assessed for damage. The skin’s thickness measured 0.55 mm. The skin was hydrated in phosphate buffer for approximately 60 min at room temperature and visually inspected for any physical defects or air bubbles. Then the skin was mounted to the Franz cell, with the stratum corneum oriented towards the donor compartment and the dermal side directed towards the receiver compartment of the diffusion cell assembly. The effective diffusion area in this configuration is 1.5 cm². The receptor compartment contained a phosphate buffer solution with a pH of 7.4. The solution was agitated at 300 rpm and maintained at 37 ± 0.5 °C during the experiment. The membrane of the donor compartment was covered with hydrogel. Small aliquots of the 1.5 mL sample were extracted and substituted with an equivalent volume of diffusion medium at 1, 2, 3, 5, 7, 10, and 12 h. Sample absorption was measured by HPLC at 321 nm using already validated method^19^. The skin permeation rate (flux, J) was determined from the slope of the linear region, alongside the calculation of other permeation parameters such as lag time (t) and the permeability coefficient for the hydrogel^26^.

#### Thermodynamic stability

Centrifugation and freeze/thaw cycle was used to examine the thermodynamic stability of the optimized FA-NE hydrogel (HG5) formulation. The durability of interfacial film of hydrogel during centrifugation shows its strength. The hydrogel was enclosed in a tightly sealed test tube and centrifuged at 3500 rpm for 30 min. The tubes were kept upright at -20˚C for 16 h and then at 25˚C for 8 h. Three freeze/thaw cycles were done, and the gel was observed for changes^27^.

#### Drug-excipients compatibility study

The FTIR studies were performed to detect any drug excipient and excipient-excipient interaction. FTIR was done on FA, all excipients, the optimized NE and hydrogel. The FTIR measurements were conducted in range of 4000–450 cm^− 1^ by using FTIR (Shimadzu FTIR)^19^

#### Stability studies

For stability studies, the hydrogels were kept in Accelerated (SC-06, Dawn Analytical Suppliers and Calibrators) and real time (SC-08, Dawn Analytical Suppliers and Calibrators) stability chamber for 6 months at Allmed Pharmaceutical Pvt. Ltd. Stability testing was conducted according to international council for harmonization (ICH) Q1A(R2) guidelines. Samples were evaluated at 0, 3 and 6 months and checked for their transparency, pH and phase separation^28^.

### In-silico analysis of ferulic acid

Molecular docking of FA with IL-17 A, IL-23, IL-6, TNF, alpha, and Ki-67 was performed by obtaining the 3D crystal structures of interleukin 6 (PDB ID: 1ALU)^29^, human TNF-α (PDB ID: 7JRA)^30^, interleukin 17 A (PDB ID: 7AMA)^31^, interleukin 23 (PDB ID: 3DUH)^32^ and Ki-67 (PDB ID: 5J28)^33^ from the RCSB Protein Data Bank. The protein structures were processed using the Protein Preparation Wizard in Schrödinger Suite. This involved assigning bond orders, adding hydrogen atoms, forming disulfide bonds, removing water molecules beyond 5Å, and predicting ionization states with the Epik tool. The missing chains and loops were repaired using the Prime module, non-active site chains were trimmed, and water orientation and protonation states were refined at pH 7.0 using PROPKA. A final energy minimization was performed with the OPLS-2005 force field to achieve an RMSD of 0.30Å for heavy atoms^34^. The 2D chemical structure of FA was retrieved from the PubChem database. The structure was processed by employing the LigPrep module in Schrödinger Suite, where it is optimized for chirality and hydrogen atoms, and minimized with the OPLS-2005 force field to yield low-energy conformers with an RMSD of 1.8Å ^35^. The most favorable active sites were selected by specifying the amino acid residues of the active binding pocket for grid generation. Finally, molecular docking was performed using the Glide module in extra precision (XP) mode with the OPLS-2005 force field. Ligands were evaluated based on docking scores that considered interactions like hydrogen bonding, electrostatic interactions, and results were visualized using the XP visualizer tool^36,37^.

### In-vivo analysis of HG5 ferulic acid nanoemulsion-based hydrogel

#### Ethics statement

The animal experiments were carried out in compliance with LCWU Ethics Institutional Review Board, referencing TDR/PRD/ETHICS/2000.1 “Operational Guidelines for Ethics Committee that Review Biomedical Research” by WHO Geneva 2000, page 5 (4.5.2), WHO rules and recommendations for biologics and ARRIVE 2.0. The study received ethical approval under the reference number ORIC/LCWU/360.

#### Experimental animals

Male Wistar rats (100–150 g), aged 6 to 7 weeks were housed in polypropylene cages at the animal house in the Institute of Pharmacy, LCWU. The rats were maintained at a temperature of 26 ± 3 °C and subjected to a 12-hour light/dark cycle. The animals were given standard pellet diet and had unrestricted access to water. There was total twenty-five rats allocated by simple randomization into five groups (*n* = 5), normal group (received no treatment), a disease group (received 5% IMQ application for 7 days), a treatment group (treated with FA-NE based hydrogel 10 mg/kg for 14 days after psoriasis induction with IMQ), a placebo group (NE based hydrogel without FA applied to IMQ treated skin for 14 days), and a standard group (topical Eczemus (Tacrolimus) 0.03% ointment applied to IMQ treated skin for 14 days)^38^. For psoriasis like model induction, same protocol was followed as employed by ^39^ on the dorsal skin of the experimental animals for 7 consecutive days. There are number of studies proved that the application of IMQ 5% on the dorsal skin of rats induces psoriasis like skin inflammation^40,41^. Similarly, the dose (i.e. 62.5 mg/kg having 3.125 active compound) has been empirically determined and previously shown in multiple publications to reliably induce optimal skin inflammation in this animal ^39,42,43^. Dorsal skin of all groups was depilated, four groups were treated topically with 5% IMQ cream every 24 h for a period of 7 days and psoriasis induction was assessed visually with psoriasis area severity index (PASI) presented in Table 4, Three parameters were assessed i.e. erythema (redness), desquamation (scale), and induration (thickness) on the dorsal skin of animals.

**Table 4:** Psoriasis Area Severity Index (PASI) Scoring.

Score	0	1	2	3	4
Erythema (Redness)	No redness	Light red	Red	Dark red	Purplish red
Desquamation (Scale)	No scale	Minor scaling present in certain lesions	Some lesions covered with spoty scaling	All lesions are scaly and patchy skin	All lesions are covered with thick stratified scaly layer
Induration (Thickness)	No lesion	Lesions protrude over healthy skin.	Lesions moderately elevated on the skin and bulging	Lesions clearly hypertrophic and bulging	Lesions are highly thick and bulging

Ref.^40^.

These three parameters were scored independently and ranging from 0 to 4, where 0 indicated no clinical sign, 1 to 4 scaling represented modest, moderate, marked, and very marked clinical signs respectively.

#### Assessment of in-vivo biomarkers and histopathological analysis

At the end of 14 days treatment schedule rats were fasted overnight and given anesthesia by injecting a mixture of xylazine and ketamine (0.2 mL/kg) into their peritoneal cavity. The mixture consisted of 2.5 mL of xylazine (50 mg/mL) and 10 mL of ketamine^44^. The responses were assessed after anesthesia to verify the state of consciousness. After confirming anesthesia, rats were euthanized by performing cardiac puncture to collect blood and dorsal skin for later study. The dorsal skin was preserved in a 10% formalin solution. Their spleens were extracted and subsequently measured in terms of weight. The spleen index was determined using the following formula^40^.

$${\mathrm{Spleen}}\;{\mathrm{Index}}\;({\mathrm{mg/g}}) = {\mathrm{Spleen}}\;{\mathrm{Weight}}\;({\mathrm{mg}})/{\mathrm{Rat}}\;{\mathrm{Weight}}\;({\mathrm{g}})$$

The enzyme linked immunosorbent assay (ELISA) kits were used to determine the inflammatory markers (IL-6, IL-17-A, IL-23, TNF-α) and biomarkers associated with oxidative stress, such as SOD, GSH, MDA, and CAT, following the instructions provided in literature with each ELISA kit by the manufacturer. The optical density (OD) was measured using a microplate reader and determined based on the methods mentioned in the kit’s literature provided by manufacturers.

For histopathological analysis the skin sample was submerged in paraffin and then sliced into sections that were 4–5 μm thick. Subsequently, the section underwent hematoxylin/eosin (H&E) staining. The histological changes were evaluated, using a light microscope with a power lens of 10 × 0.25 mm ^34^. Immunohistochemical slides were developed using formalin-preserved tissue specimens to detect the presence of Ki-67 protein. The antigen-antibody combination was seen using DAB chromogen ^45^.

### Statistical analysis

The data was expressed as the mean ± standard deviation (SD) and subjected to statistical analysis using one-way analysis of variance (ANOVA) followed by Dunnett’s post hoc test, performed with GraphPad Prism 9. A p-value less than 0.05 was deemed statistically significant.

## Results

### Assessment of oils, surfactants, and cosurfactant for nanoemulsion development

The drug solubilizing capacity in oil phase is key attribute, as it significantly determines the drug loading capacity of formulation. The FA solubility was assessed in three different oils and it was found that the solubility of FA was 5.0 ± 0.48 mg/mL in castor oil 3.31 ± 0.27 in Olive oil and 2.58 ± 0.24 in ISIS. Based on solubility study castor oil was selected for further NE formulation as oil phase. For less toxic NE development nonionic surfactant in low concentrations are preferably used over ionic surfactants^16^. Further, Tween-80 is extensively utilized as an emulsifier and is safe for human skin at concentrations up to 15%^46^. For this purpose, the solubility of FA was assessed in Tween-80 and laberasol. Solubility of FA was greater in Tween 80 (77.70 ± 2.17) as compared to in laberasol (48.63 ± 0.74), hence tween 80 was selected as surfactant for further studies. Then solubility also assessed in cosurfactants, propylene glycol and glycerin, where propylene glycol was selected due to high solubility (47.07 ± 1.11) as compared to in glycerin (20.57 ± 0.55) (Table 5). Propylene glycol has been employed as an emollient and humectant, with a safe concentration for topical formulations of up to 50%^47^.

**Table 5:** Solubility study of Ferulic Acid in different oils, Smix (Surfactant & Co- surfactant).

Sr. No	Component	Solubility (mg/ml)
1.	Oils	
1.1	Castor Oil	5.03 ± 0.48
1.2	Olive Oil	3.31 ± 0.27
1.3	Isostearyl Isostearate ISIS	2.58 ± 0.24
2	Sufactant	
2.1	Tween-80	77.70 ± 2.17
2.2	Labrasol	48.63 ± 0.74
3	Co-sufactant	
3.1	Propylene Glycol	47.07 ± 1.11
3.2	Glycerine	20.57 ± 0.55

Values expressed as mean ± SD (*n* = 3).

### Pseudo-ternary phase diagrams study

Accurate ratios of surfactants and cosurfactants are crucial for the successful and stable formation of NEs. A 1:1 ratio of surfactant to cosurfactant (Smix) produced a significant NE region, indicating successful interfacial tension reduction and improved stabilization of the oil-water interface. This balanced ratio likely enhances the efficient packing of the interfacial film, encouraging the spontaneous generation of nano-sized droplets. Augmenting the cosurfactant concentration (Smix 1:2) substantially enlarged the NE domain, presumably due to the enhanced flexibility of the interfacial film, enabling the system to support a broader spectrum of compositions. A greater surfactant-to-cosurfactant ratio (Smix 2:1) produced a diminished NE zone. The diminished amount of cosurfactant may increase the rigidity of the interfacial film, hence limiting the generation and stabilization of nano-sized droplets throughout a wider compositional spectrum. Pseudo-ternary phase diagrams were constructed using different proportions of castor oil without the inclusion of drug. The optimal NE zones were identified at oil: Smix ratios of 5:5 and 6:4. (Fig. 1) These two ratios were selected for further investigation and preparation of drug-loaded NEs.

### Characterization of optimized ferulic acid nanoemulsion formulations

#### Particle size, polydispersity index, zeta potential and entrapment efficiency, pH

The particle size of all NEs ranges from 29.8 ± 0.49 to 43.5 ± 0.45 nm. The formulation with the smallest particle size was NE5. The PDI ranges from 0.21 ± 0.02 to 0.31 ± 0.01 showing uniformity of droplet size distribution and homogeneity of NE, while the zeta potential and pH vary from 28.5 ± 0.57 to 32.7 ± 0.31 and 5.5 ± 0.8 to 6.8 ± 0.3, respectively. The entrapment efficiency varies from 90.48 ± 0.31 to 96.26 ± 0.33. The results of all these parameters are presented in Table 6. The dispersion stability analysis revealed the absence of phase separation, cracking, creaming, and coalescence in the formulations. The NE5 was utilized for the manufacture of hydrogels due to its smaller particle size and good entrapment efficiency.

**Table 6:** Particle Size, Polydispersity Index, Zeta potential, Entrapment efficiency and pH of ferulic acid nanoemulsions.

Formulation	PS (nm)	PDI	Z.*P* (mV)	Entrapment Efficiency (%)	pH
NE 1	40.2 ± 0.15	0.31 ± 0.01	28.4 ± 0.47	95.24 ± 0.48	6.4 ± 0.31
NE 2	31.0 ± 0.60	0.23 ± 0.02	31.0 ± 0.70	94.90 ± 0.12	6.1 ± 0.15
NE 3	30.8 ± 0.85	0.21 ± 0.02	30.5 ± 0.46	95.58 ± 0.35	6.2 ± 0.17
NE 4	33.1 ± 0.91	0.25 ± 0.02	31.0 ± 0.60	91.84 ± 0.21	6.6 ± 0.26
NE 5	29.8 ± 0.49	0.21 ± 0.02	28.5 ± 0.57	96.26 ± 0.33	6.8 ± 0.15
NE 6	43.5 ± 0.45	0.27 ± 0.01	32.7 ± 0.31	90.48 ± 0.31	5.5 ± 0.26

PS=particle size, PDI=polydispersity index, z.p= zeta potential. All results are presented as Mean ± SD (*n* = 3). Number of runs per sample is 3.

#### Dispersion stability

The examination of dispersion stability revealed no evidence of phase separation, cracking, creaming, or coalescence in the formulations. Moreover, NE5 showed excellent dispersion stability, evidenced by a minimal droplet size (nm), a low polydispersity index, and a high zeta potential (mV), with no detectable phase separation during storage. The results of all these parameters are presented in Table 7.

**Table 7:** Particle Size, Polydispersity Index (PDI) and Particle Charge (Zeta Potential), after dispersion stability.

Formulation	PS (nm)	PDI	Z.*P* (mV)
NE 1	39.87 ± 0.42	0.35 ± 0.05	28.8 ± 0.59
NE 2	31.32 ± 0.62	0.26 ± 0.04	31.2 ± 0.89
NE 3	33.07 ± 0.60	0.28 ± 0.05	30.8 ± 0.25
NE 4	33.17 ± 0.65	0.27 ± 0.05	31.6 ± 0.35
NE 5	29.93 ± 0.68	0.26 ± 0.03	28.7 ± 0.49

PS=particle size, z.p= zeta potential, PDI=polydispersity index. All results are presented as Mean ± SD (*n* = 3) (Number of runs per sample were 3).

### Optimization of nanoemulsion based hydrogel

#### Rheology, pH & spreadability

**Fig. 2:**
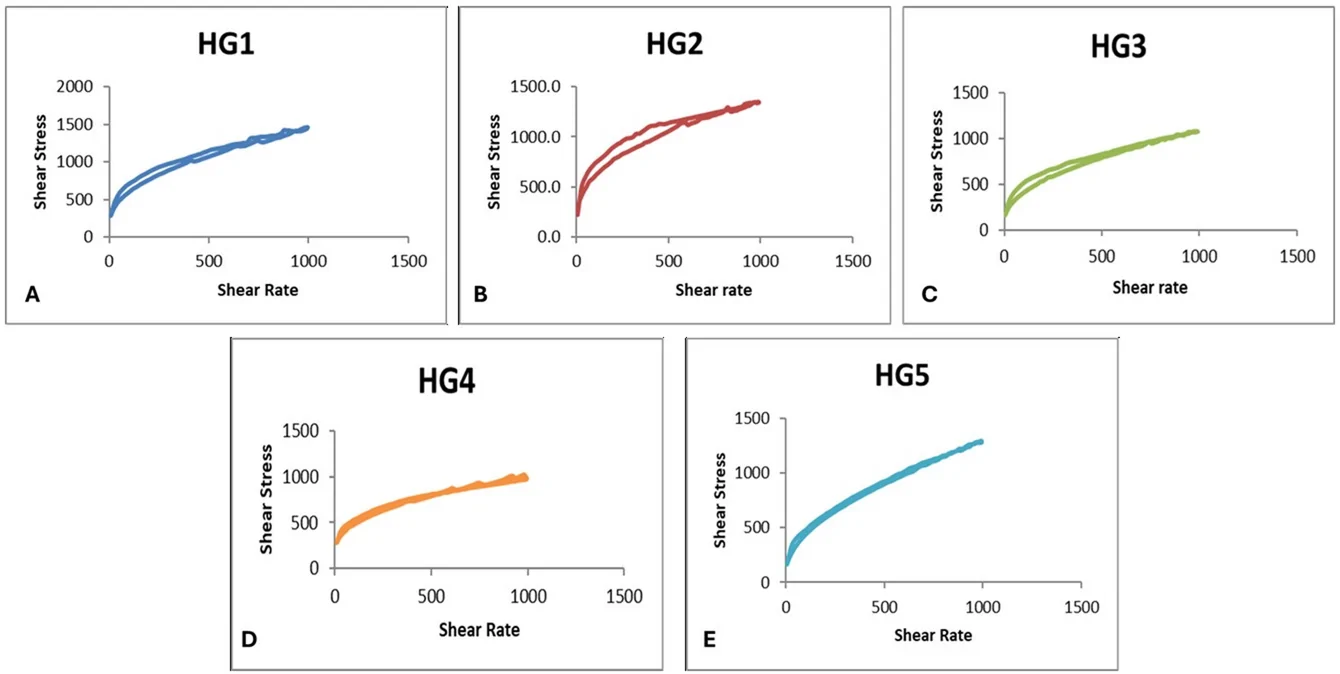
Rheograms of Nanoemulgels after 24 h. Data represents the upward and downward flow curves used to determine yield value and flow behavior. (**A**) HG1, (**B**) HG2, (**C**) HG3, (**D**) HG4, (**E**) HG5.

The rheograms of all formulations were obtained 24 h following the manufacture of the NEs. All rheograms displayed a hysteresis loop (Fig. 2) indicative of plastic and pseudoplastic (shear-thinning) behavior, accompanied by thixotropy. The presence of hysteresis loops in the rheograms of all nanoemulgels after 24 h was a distinctive characteristic of the materials. The existence of the hysteresis loop signifies a breakdown in gel structure, and the area enclosed by the loop may serve as an index for the extent of breakdown or thixotropy.

The yield values of formulations HG1, HG2, and HG4 were greater (474.2, 492.6, and 394.5 Pa, respectively) compared to HG3 and HG5, which had values of 314.3 and 325.5 Pa, respectively (Table 8). These nanoemulgels exhibited distinct yield values, indicating the crucial force necessary to disrupt the internal gel structure prior to the onset of flow. When the applied stress surpasses the yield point, the material commences to flow, demonstrating a reduction in viscosity as the shear rate increases. The apparent viscosities of formulations HG1, HG2, and HG4 were elevated (18.11, 17.88, and 15.04 Pa.s, respectively) in comparison to the apparent viscosities of formulations HG3 and HG5 (11.98 and 11.36 Pa.s, respectively) at a shear rate of 990 1/s, as shown in Table 8. Elevated apparent viscosities may result from enhanced interaction forces and the asymmetric molecular structure, which raises viscosity and exhibits resistance to flow^23^.

**Table 8:** Physiochemical properties of hydrogel formulations.

Formulations	Yield Value (Pa)	Apparent Viscosity at ShearRate 990 (1/s) (Pa s)	pH	Spreadability (gcm/s)
HG1	474.2	18.11	5.5 ± 0.5	5.4 ± 1.26
HG2	492.6	17.88	6.3 ± 0.92	6.8 ± 0.81
HG3	314.3	11.98	6.0 ± 0.26	7.7 ± 1.43
HG4	394.5	15.04	6.4 ± 0.66	6.6 ± 1.1
HG5	325.5	11.36	6.2 ± 0.15	8.2 ± 0.89

Results are presented as Mean ± SD (*n* = 3).

#### In-vitro skin permeation study of a ferulic acid nanoemulsion-based hydrogel

The permeation parameters exhibited distinct variations among the formulations, underscoring the impact of formulation composition on drug diffusion behavior. After 12 h, the penetration of drug was markedly superior in HG5 (432.64 ± 2.65 µg/cm²) (Table 9) compared to the other formulations. This is due to the small droplet size and low viscosity of NE5 utilized in the FA-NE hydrogel formulation. The additional permeability data (Table 10) are likewise notably favorable for HG5, with the steady-state flux being the highest for HG5 at 32.0 ± 0.36 µg/cm²/hr. The minimal lag time (0.82 ± 0.05) suggests that HG5 exhibits a rapid commencement of permeation relative to other hydrogels, while the elevated Permeability Coefficient for HG5 further corroborates its enhanced drug transport across the membrane. HG5 was determined to be the optimal formulation, providing quick onset and improved drug penetration.

**Table 9:** Percentage Permeation of FA of all the Hydrogel formulations.

Time	HG1 (µg/cm²)	HG2 (µg/cm²)	HG3 (µg/cm²)	HG4 (µg/cm²)	HG5 (µg/cm²)
1 h	26.96 ± 0.10	18.48 ± 1.93	48.16 ± 0.20	46.0 ± 0.48	51.12 ± 0.37
2 h	44.32 ± 0.21	54.8 ± 0.13	69.92 ± 0.42	58.56 ± 0.44	69.28 ± 0.43
3 h	72.40 ± 0.31	77.92 ± 0.26	148.88 ± 0.59	96.16 ± 1.77	144.48 ± 1.84
5 h	118.8 ± 0.64	155.36 ± 0.69	200.24 ± 0.99	126.0 ± 5.33	212.8 ± 2.13
7 h	178.48 ± 0.56	221.2 ± 0.64	280.56 ± 2.93	182.88 ± 3.55	293.04 ± 3.45
10 h	260.72 ± 0.42	277.84 ± 2.29	299.84 ± 5.55	225.6 ± 4.31	396.48 ± 2.67
12 h	351.28 ± 2.86	321.92 ± 0.74	366.0 ± 3.21	338.24 ± 1.76	432.64 ± 2.65

Values are expressed as Mean ± SD, *n* = 3.

**Table 10:** Determination of permeation parameters including flux, lag Time and permeability coefficient.

Gel	Flux (J) (µg/cm²/h)	Lag Time (t) (h)	Permeability Coefficient (Kp) (cm/h)
HG1	30.99 ± 0.31	0.90 ± 0.10	0.00108 ± 0.0014
HG2	27.11 ± 0.11	1.05 ± 0.07	0.00095 ± 0.0051
HG3	24.13 ± 0.42	1.29 ± 0.06	0.00084 ± 0.0031
HG4	26.9 ± 0.32	1.01 ± 0.05	0.00094 ± 0.0012
HG5	32.02 ± 0.36	0.82 ± 0.05	0.00112 ± 0.0038

Values are expressed as Mean ± SD, *n* = 3.

#### Stability studies of HG5 hydrogel containing ferulic acid nanoemulsion over a period of six months

The chosen HG5 formulation underwent physical stability testing, revealing that it remained stable for six months at storage condition of temperature 25 °C ± 2 °C, relative humidity 60% ± 5% and at temperature 40 °C ± 2 °C, relative humidity 75% ± 5% in HDPE bottle with child resistant lid. The formulation exhibited no phase separation. The formulation remained transparent, and the pH was maintained between 6 and 6.4 during a period of six months. All findings are displayed in Table 11.

**Table 11:** Stability of HG5 ferulic acid nanoemulsion based hydrogel.

Time	Temp. & RH	Transparency	pH	Phase separation
0 month	25 °C ± 2 °C 60% ± 5%	+	6.0 ± 0.2	–
	40 °C ± 2 °C, 75% ± 5%	+	6.4 ± 0.3	–
3 months	25 °C ± 2 °C 60% ± 5%	+	6.3 ± 0.4	–
	40 °C ± 2 °C 75% ± 5%	+	6.3 ± 0.4	–
6 months	25 °C ± 2 °C 60% ± 5%	+	6.4 ± 0.4	–
	40 °C ± 2 °C 75% ± 5%	+	6.3 ± 0.3	–

Mean ± SD (*n* = 3); +: presence, −: absence.

### FTIR Studies of ferulic acid and the excipients

**Fig. 3:**
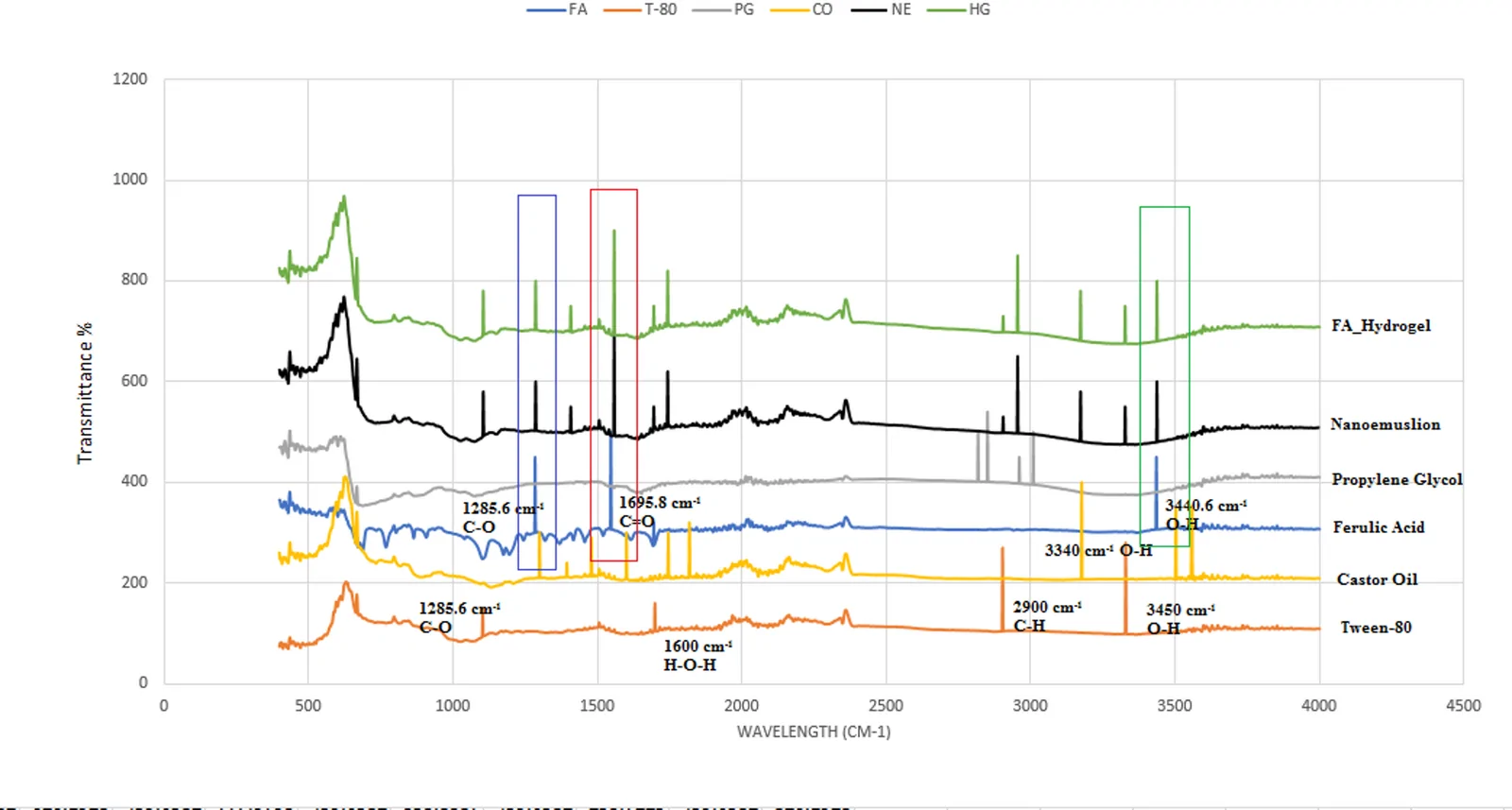
FTIR Spectrum of ferulic acid nanoemulsion and hydrogel with other excipients. FA Ferulic Acid, HG5 Hydrogel, NE5 Nanoemulsiuon, PG polyethylene glycol, CO castor oil, T-80 Tween-80.

The chemical interactions between FA and the excipients were analyzed to enhance the understanding of the compatibility of drug and excipients in formulation. The FTIR studies were conducted for FA, drug loaded NE5, castor oil, tween-80, propylene glycol and FA-NE hydrogel (HG5). The spectra obtained from FTIR study indicated sharp peak of FA was found at 3440.6 cm-1 (carboxylic acid O-H stretching), 1695.8 cm-1 (carboxylic acid C = O stretching), 1285.6 cm-1 (carboxylic acid C-O stretching); 1523 and 1625.1 cm-1 (aromatic C = C) which confirm the skeleton of FA. These peaks were still present after the NE and hydrogel formulations with only a small modification (Fig. 3), peaks in hydrogel and NE represent the functional groups of FA C-O peak (mark as purple rectangle), C = O peak (mark as red rectangle) and O-H peak (mark as green rectangle)^48^. Similarly, the peaks of Tween-80 and castor oil could be clearly observed in NE and FA-NE hydrogel formulation with minor modifications. This demonstrated that FA was acceptable when combined with other excipients in formulations.

### Morphological analysis by scanning electron microscope

**Fig. 4:**
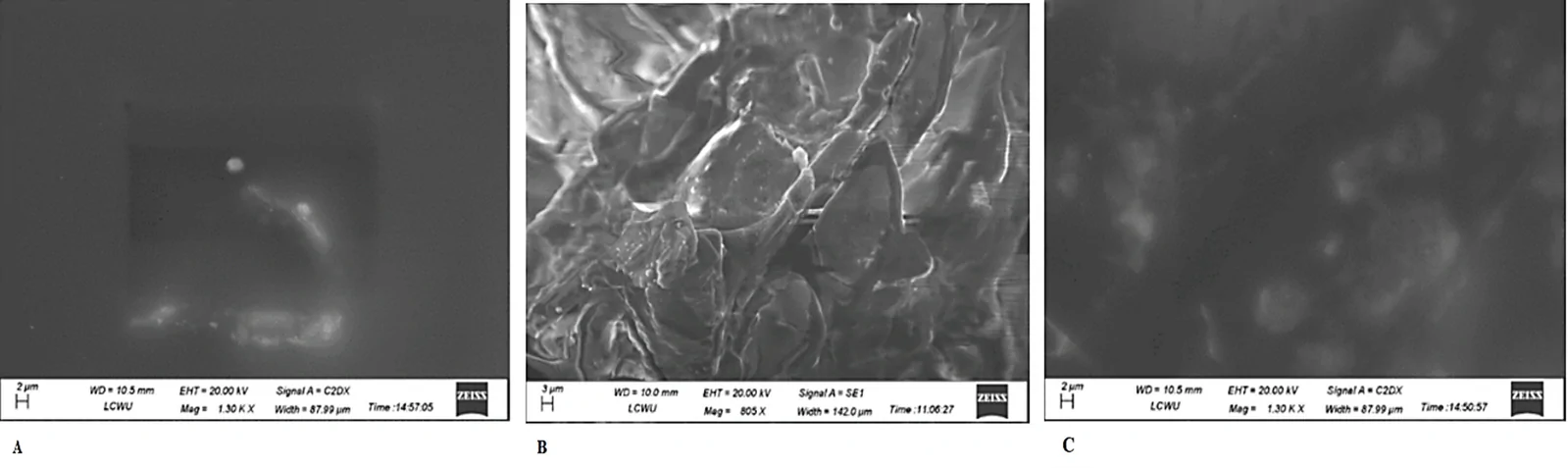
Scanning electron microscopy of ferulic acid loaded hydrogel. (**A**) Nanoemulsion, (**B**) Pure drug (Ferulic acid) and (**C**) FA-NE hydrogel (HG5).

SEM was employed to investigate the morphology of the formulations (Fig. 4).

Figure 4A shows dispersed bright particulates within a darker matrix, likely corresponding to concentrated or solidified oil droplets, aggregated nanosized droplets, or solute residues formed upon drying. Figure 4B reveals a rigid, dehydrated solid lacking soft-matrix artifacts, representing the crystalline morphology of the pure drug. Figure 4C depicts a hydrated, insulating polymer network in which fine pore walls appear partially collapsed or obscured; the hydrogel’s low electron yield and possible retained moisture or charging suggest that an internal porous network is present but not well resolved under the selected imaging mode and pressure.

### In-silico docking of Ferulic acid

**Fig. 5:**
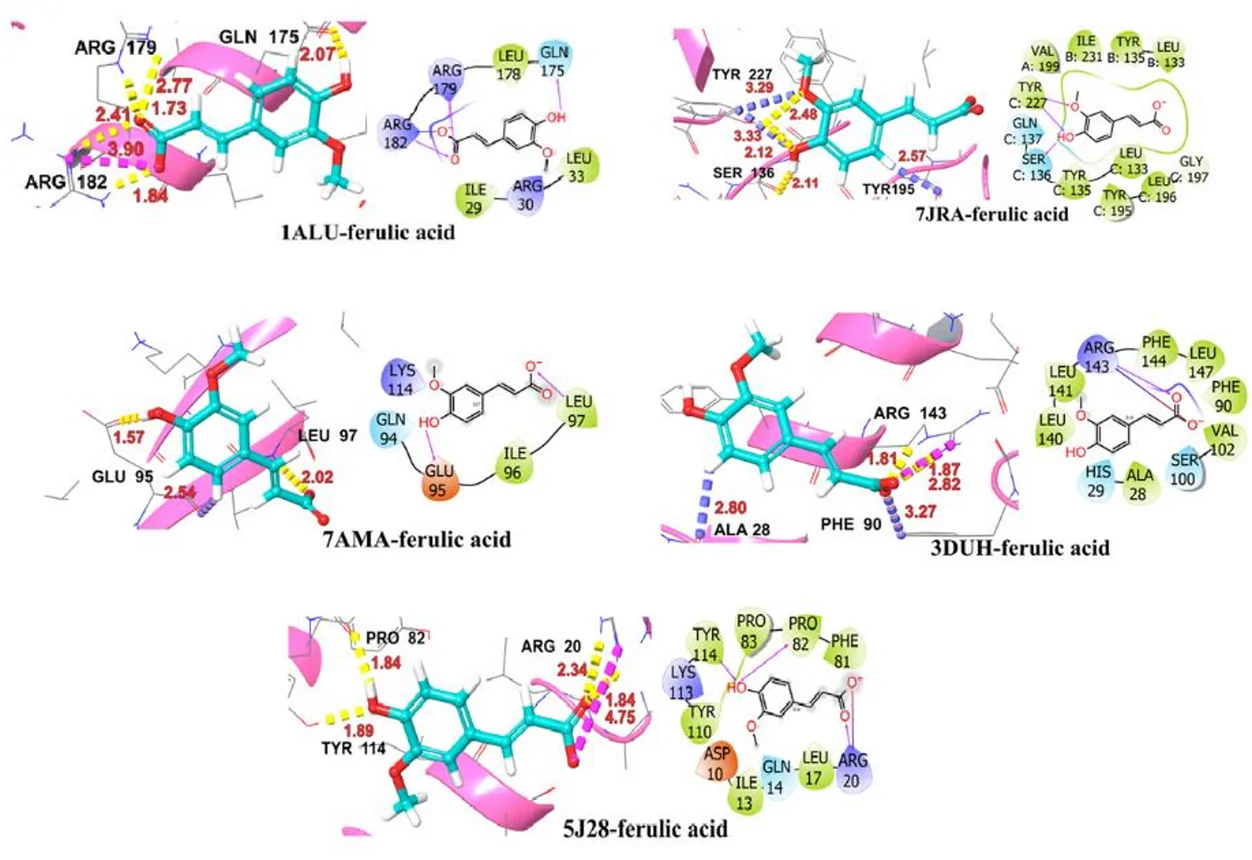
2D and 3D docking interactions of ferulic acid with target proteins. 1ALU (IL-6), 7JRA (TNF-α), 7AMA (IL-17 A), 3DUH (IL-23), 5J28 (Ki-67).

Figure 5 depicts the 2D and 3D interactions of FA within the binding pocket of target proteins, including 1ALU (IL-17 A), 7JRA, 7AMA, 3DUH, and 5J28. The FA was docked into the defined binding pockets of selected targets, revealing favorable binding energies in the range of -4.1 to -6.6 kcal/mol. The superior glide scores of -6.6 kcal/mol were observed with TNF-α (7JRA), attributed to conventional H-bonding interactions with amino acid residues such as TYR227, SER136, and π-hydrogen bonding interactions with residues including TYR227, TYR195. The higher binding score and substantial H-bonding interactions with key amino acid residues of 7JRA indicate the stability of FA conformation within the binding pocket. The p19 subunit (TRP26, ARG159, TYR115, and PRO116) of 3DUH was explored for the anti-inflammatory effect of FA. The FA formed four to five conventional- and π-hydrogen bonds with amino acid residues of 3DUH, showing a docking score of -4.9 kcal/mol. The carboxylate group of FA showed ionic interactions with the basic amino acid residue ARG143, but no interaction was observed with key amino acid residues. The dimer interface of 7AMA is the primary binding site of small molecules. The interaction with key amino acids, including LEU97, ASN88, TYR85, and VAL98, resulted in disruption of structural integrity, a prerequisite for proper functioning of 7AMA (IL-17 A). FA displayed only one hydrogen bonding interaction with key amino acid LEU97, thereby indicating lower affinity with docking scores of -4.1 kcal/mol. Similarly, FA demonstrated G-scores of -4.1 and − 4.3 kcal/mol with 1ALU and 5J28, respectively, indicating its lack of interaction with any of the key amino acid residues within the binding pocket. These findings underscore the potential of FA to act as an anti-inflammatory agent, attributed to significant TNF-α docking results, offering a detailed molecular basis for further drug optimization. The details of docking scores, E-model scores, and key interactions of FA against target proteins are outlined in Table 12. The 2D and 3D interactions are provided in Fig. 5.

**Table 12:** Molecular Glide score, Hydrogen bonding and ionic interactions with distances (Å) for ferulic acid with target proteins including 1ALU, 7JRA, 7AMA, 3DUH, and 5J28.

Compounds	G-Score (kcal/mol)	Emodel	H.B.I Residue (Distance Å)	Ionic & π-H bonding interaction
1ALU (IL-6)				
Ferulic acid	− 4.1	− 32.30	GLN175 (2.07) ARG179 (1.73) ARG179 (2.77) ARG182 (1.84) ARG182 (2.41)	ARG182 (3.90)
7JRA (TNF-α)				
Ferulic acid	− 6.6	− 45.28	TYR227 (2.48) TYR227 (2.12) SER136 (2.11)	TYR227 (3.29) TYR227 (3.33) TYR195 (2.57)
7AMA (IL-17 A)				
Ferulic acid	− 4.1	− 25.29	GLU95 (1.57) LEU97 (2.02)	GLU95 (2.54)
3DUH (IL-23)				
Ferulic acid	− 4.9	− 30.83	ARG143 (1.81) ARG143 (1.87)	ALA28 (2.80) PHE90 (3.27) ARG143 (2.82)
5J28 (Ki-67)				
Ferulic acid	− 4.3	− 29.49	ARG20 (1.84) ARG20 (2.34) PRO82 (1.84) TYR114 (1.89)	ARG20 (4.75)

H.B.I: Hydrogen bonding interacting residues.

### Results of analysis of biomarkers of psoriasis

#### Psoriasis Area Severity Index (PASI)

**Fig. 6:**
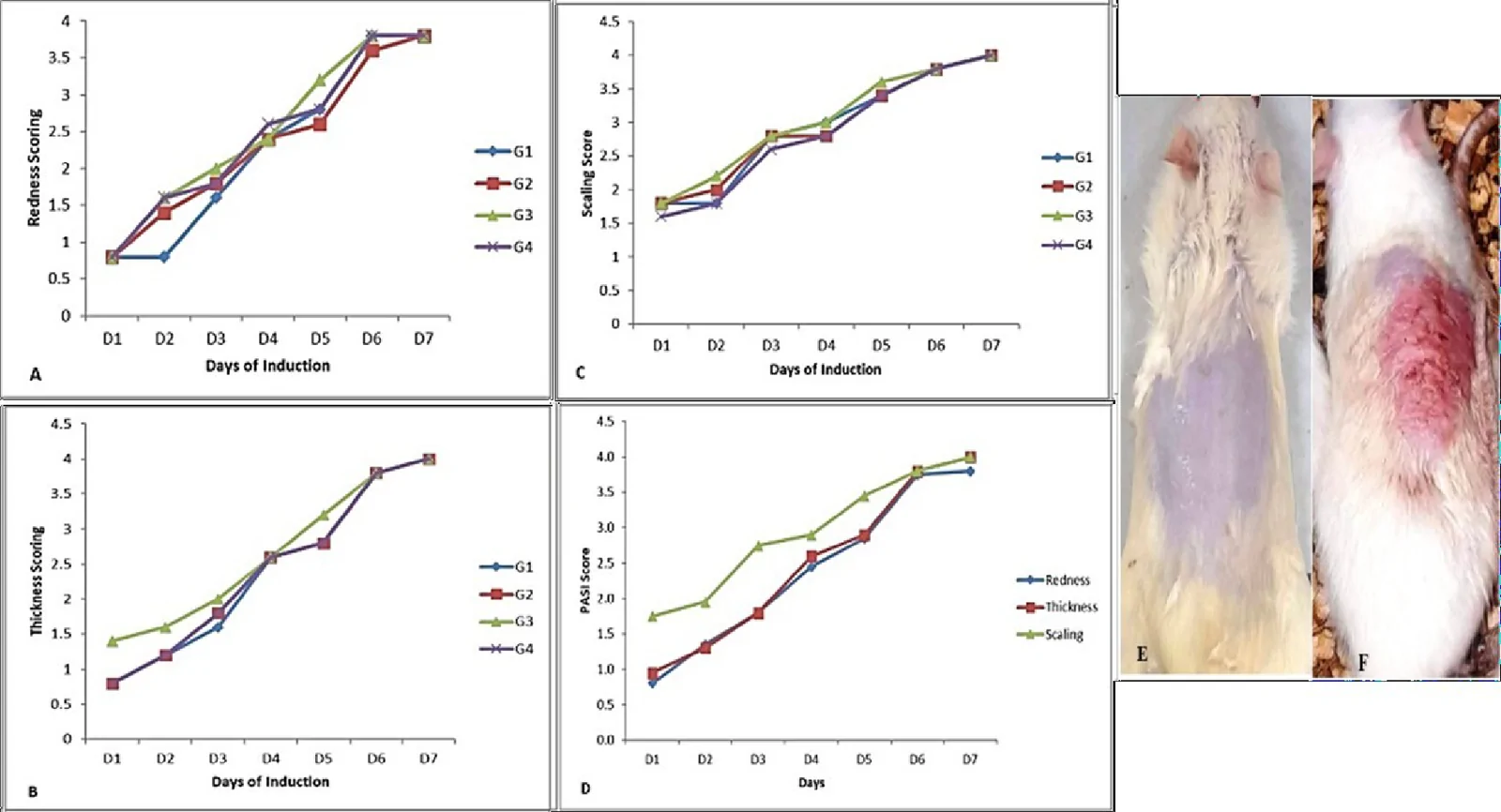
Evaluation of Psoriasis Area Severity Index (PASI) and macroscopic presentation of dorsal skin before and after application of IMQ 5% cream for 7 days. (**A**) Redness score; (**B**) Thickness score; (**C**) Scaling score; (**D**) cumulative PASI score; (**E**) Rat dorsal skin at day 1; (**F**) Rat dorsal skin at day 7.

The PASI score was determined by the visual assessment of rat skin from day 1 to day 7, as parameters are outlined in Table 4. The scores for redness, thickness, and scaling in the four groups treated with IMQ are illustrated in Fig. 6 (A, B, C, and D). The redness score of all groups on day 1 after IMQ application was 0.8, thickness score was 1 and scaling score was 1.8 and on the 7th day the redness score increased to 3.8 while both the thickness and scaling score rose to 4. Figure 6E & F illustrates the visual presentation confirming the development of psoriatic lesions. After treatment with FA-NE hydrogel on day 14, the redness, thickness, and scaling scores of the treatment group diminished to 0.2 which is quite similar to standard group, while in the disease and placebo group, the redness, thickness, and scaling scoring was 1.4, 1, and 1.8, respectively.

#### Effect of ferulic acid hydrogel treatment on psoriatic skin, inflammatory and oxidative stress markers

**Fig. 7:**
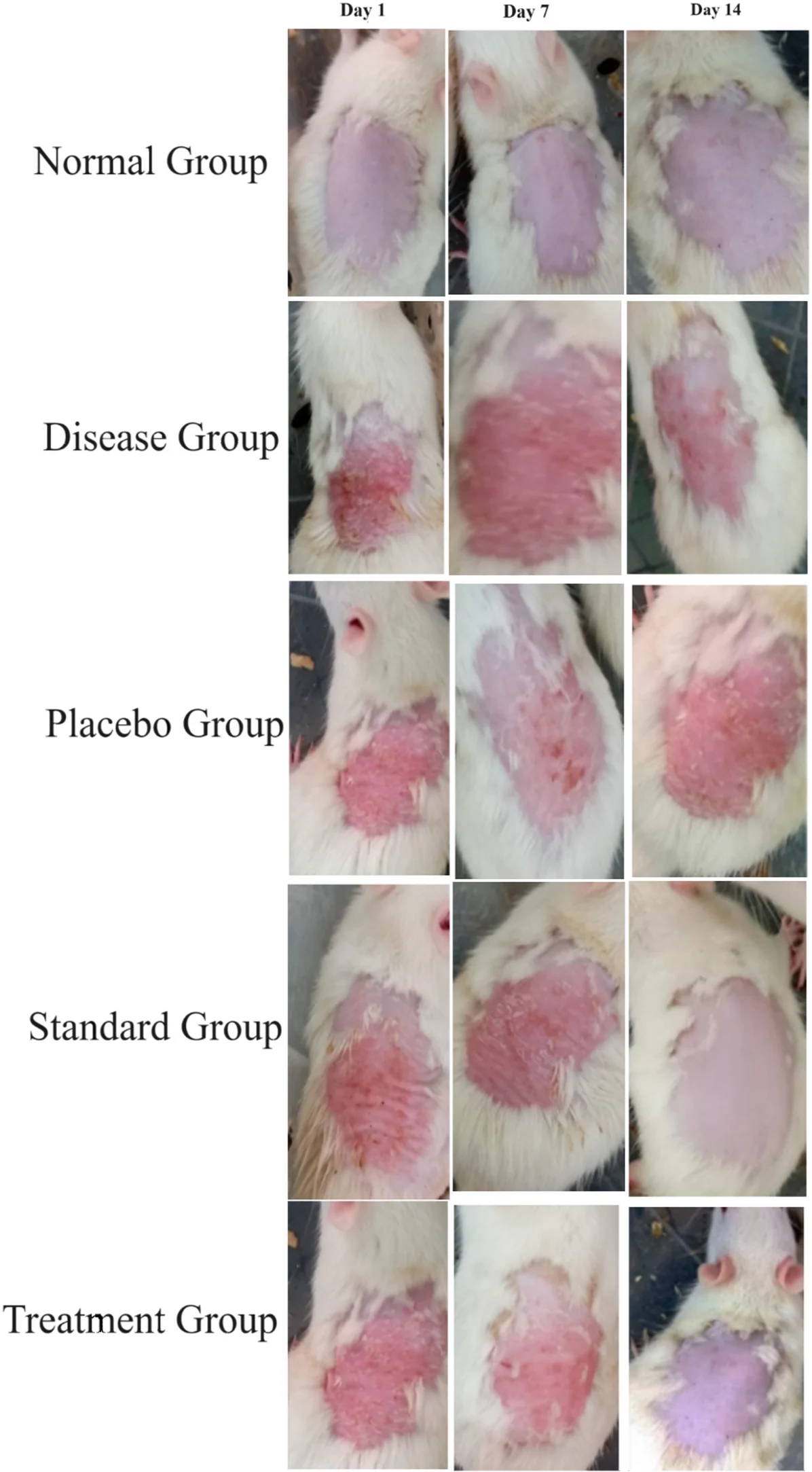
Photographic images of dorsal skin of all experimental groups at 1st, 7th and 14th days of treatment. Normal group (received no treatment), Disease group (received 5% IMQ application), Placebo group (NE based hydrogel without FA applied to IMQ treated skin, Standard group (topical Eczemus (Tacrolimus) 0.03% ointment applied to IMQ treated skin, Treatment group (treated with FA-NE hydrogel 10 mg/kg for 14 days after psoriasis induction with IMQ).

The skin was visually monitored over a 14-day treatment period. Evaluation of the skin condition before treatment, after IMQ application, and following treatment with the FA-NE hydrogel indicated that it markedly relieved the symptoms of experimentally induced psoriasis. The visual depiction of the dorsal surface of rats at 1st, 7th and 14th days of treatment showed that the disease and placebo groups showed redness on 14th day whereas the skin of standard and treatment groups were entirely healed as presented in Fig. 7 these results were found to be like the effects of standard tacrolimus.

**Fig. 8:**
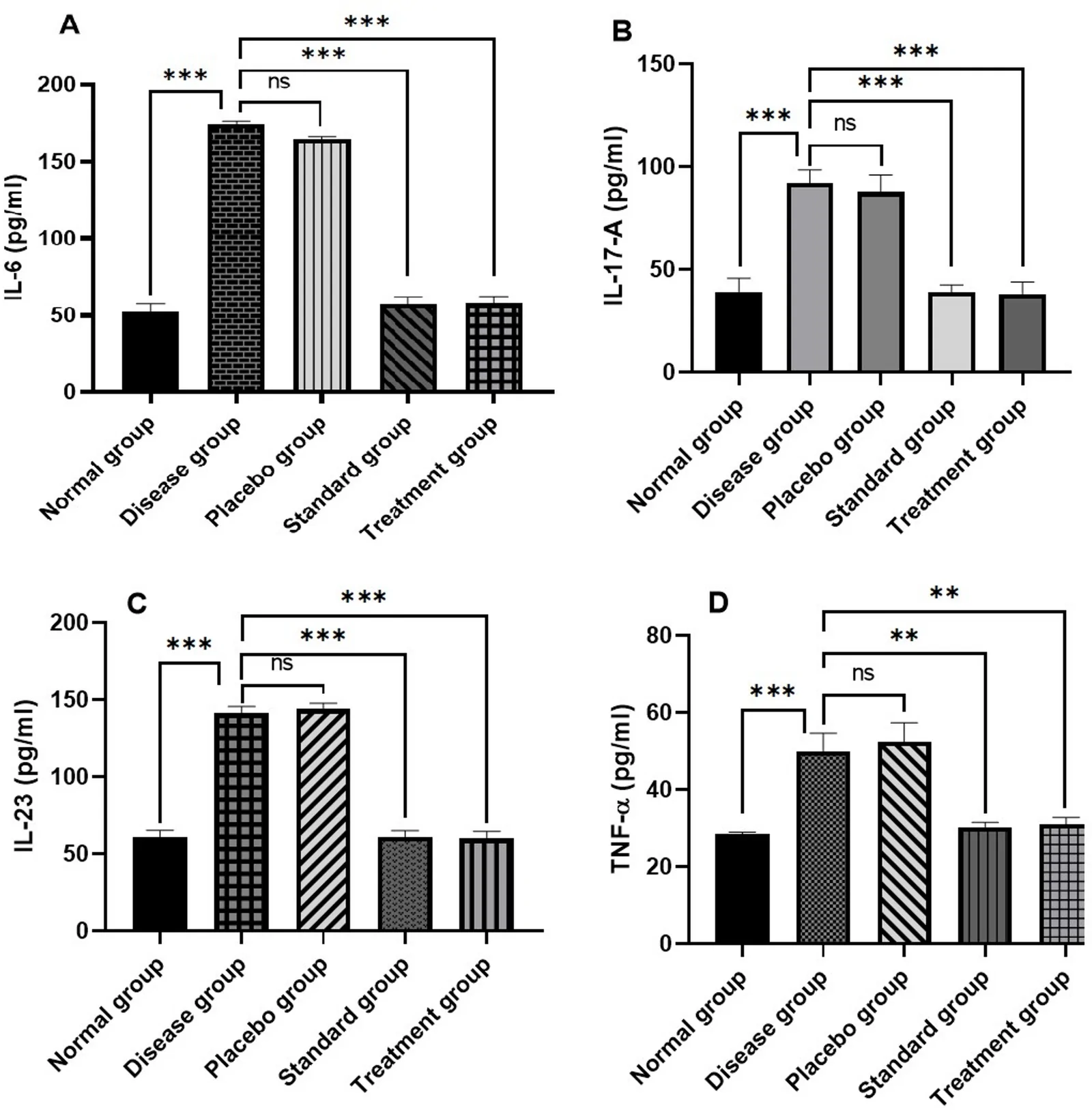
Concentration of inflammatory biomarkers in serum sample of all experimental groups. All values were expressed as mean ± SEM (*n* = 5) * represents *p* < 0.05, ** *p* < 0.01, *** *p* < 0.001 when compared with diseased group. Significant differences among treatments were observed after one way ANOVA analysis followed by Dunnett’s post-hoc test. ns represents no statistical difference.

After seven days of application of IMQ there was a considerable increase in the levels of inflammatory biomarkers, including IL-6 (174.5 ± 1.7 pg/mL), IL-17-A (92 ± 7.1 pg/mL), IL-23 (181.3 ± 2 pg/mL), and TNF-α (53.9 ± 6.8 pg/mL), but after treatment with the FAH, the inflammatory biomarkers level dropped in comparison to the disease group, and the results were found to be like those of the standard group (*p* < 0.05). The IL-6, IL-17 A, IL-23 and TNFα of the treatment group decreased to 52.9 ± 3.2, 38.1 ± 6.4, 59.8 ± 5.2 and 26.9 ± 1.3 pg/mL when compared to the disease group (Fig. 8A, B, C & D).

The levels of cytokines in treatment group were closer to the levels observed in normal group, IL-6 (52.5 ± 5.8 pg/mL), IL-17-A (30.1 ± 7.3 pg/mL), IL-23 (60.6 ± 5.1 pg/mL), and TNF-α (28.4 ± 0.7 pg/mL). There was no statistically significant difference observed between the disease group and the placebo group (*p* > 0.05). The evaluation of oxidative stress markers is vital for understanding the extent of oxidative damage within biological systems. Oxidative stress results from an imbalance between the production of reactive oxygen species (ROS) and the body’s ability to detoxify them or repair the resulting damage. The treatment of IMQ for seven days resulted in a considerable increase in MDA levels. The application of FA-NE hydrogel resulted in a significant reduction in the blood level of MDA in both the treatment and standard groups (*p* < 0.05) compared to the diseased group. However, the levels of MDA remained elevated in the disease and placebo groups (*p* > 0.05). The levels of other antioxidants such as SOD, GSH, and CAT were increased in the treatment group, like the standard group (*p* < 0.05), and drastically decreased in the disease group (Table 13).

**Table 13:** Relative Expression of Oxidative Stress Related Indicators in different experimental Groups.

Sr. No	Groups	MDA (mmol/mL)	SOD (U/mL)	GSH (µmol/mL)	CAT (U/mL)
1.	Normal group	5.8 ± 0.9***	54.4 ± 8.3***	76.4 ± 13.2***	90.4 ± 5.8***
2.	Disease group	16.9 ± 3.3	22.9 ± 1.2	33.9 ± 9.3	47.35 ± 7.4
3.	Placebo group	15.7 ± 3.9^ns^	24.4 ± 3.3 ^ns^	36.2 ± 4.27 ^ns^	48.4 ± 5^ns^
4.	Treatment group	5.4 ± 0.7***	52.8 ± 9.6***	75.9 ± 13.29***	89.1 ± 3.2***
5.	Standard group	5.4 ± 1.1***	53.7 ± 17.4***	76.6 ± 15.7***	90.4 ± 8***

All values were expressed as mean ± SD (*n* = 5) * Represents *p* < 0.05, ** *p* < 0.01, *** *p* < 0.001 when compared with diseased group. Significant differences among treatments (*p* < 0.001) were observed after analysis by one way ANOVA followed by Dunnett’s post-hoc test. ns represent no statistical difference *p* > 0.05 when compared with diseased group.

#### Spleen index analysis of spleen specimens extracted from distinct groups subjected to different pharmacological treatments

**Fig. 9:**
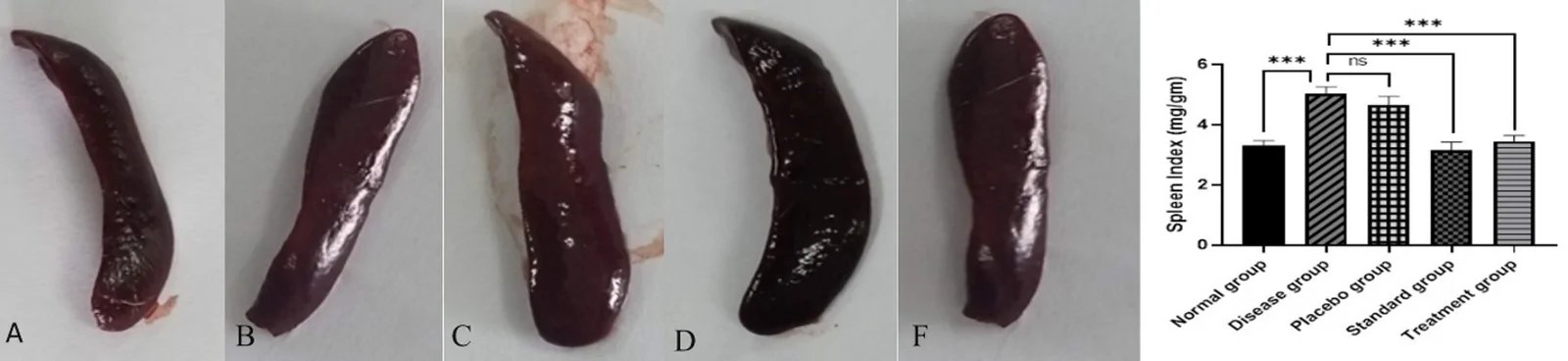
Visual presentation of spleen removed from various treated groups and graphical presentation of spleen index (mg/gm) of all experimental groups. (**A**) Normal Group, (**B**) Treatment Group, (**C**) Diseased group, (**D**) Vehicle control (Placebo) group, (**E**) Standard group.

The use of IMQ cream resulted in an increased spleen index, which was dramatically reduced by FA-NE hydrogel treatment. In the disease group, the spleen index (5.03 ± 0.2) remained considerably increased compared to the FA-NE hydrogel group (3.4 ± 0.2***) and standard group (3.1 ± 0.3***). Table 14; Fig. 9 present the spleen pictures and the index for all groups.

**Table 14:** Changes of spleen index in different experimental groups.

Sr. No	Groups	Spleen Index (mg/g)
1.	Normal group***	3.32 ± 0.3
2.	Treatment group***	3.44 ± 0.4
3.	Disease group	5.03 ± 0.5
4.	Vehicle control/placebo group ^ns^	4.64 ± 0.7
5.	Standard group***	3.14 ± 0.6

All values were expressed as mean ± SD (*n* = 5).

#### Histopathological & immunohistochemistry examination of skin excised from all groups at the end of study

**Fig. 10:**
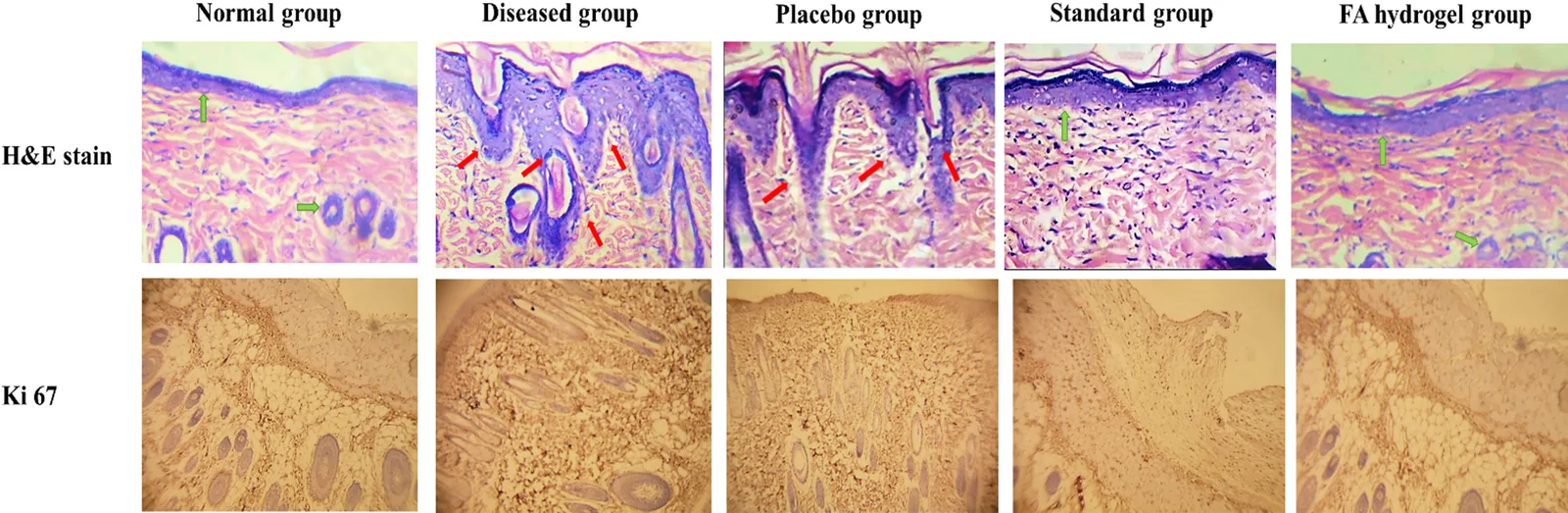
Histopathological changes in IMQ induced psoriatic lesions with different treatments (100x). The green arrow shows normal epidermis and dermis. The red arrows represent the increased thickness in epidermal layer and damage dorsal skin of rats with psoriatic lesions in diseased and placebo group respectively.

In normal rats the histological examination showed no sign of inflammatory cell infiltration in the dermis. After seven days of IMQ application, psoriasis-like lesions emerged, the epidermis with marked thick layers accompanied by parakeratosis/hyperkeratosis, no granular layer, hypertrophy of the spinous layer, and downward elongation of the epidermal rete ridges (as represented by a red arrow) illustrated in Fig. 10. These histopathological features closely resembled human psoriasis. These pathological changes were significantly ameliorated with the topical application of FA-NE hydrogel same as in standard group. While the minimal improvement was observed in the placebo group, which remained histopathologically like the diseased group.

The immunohistochemistry analyses conducted for Ki-67 for all experimental groups. Figure 10 illustrates that the distribution of Ki-67 along the ridges in psoriatic skin, both in the disease and placebo groups, is markedly dense compared to normal, standard and FA-NE hydrogel treated rats’ skin, which exhibits an absence of Ki-67. The proliferation rate of keratinocytes is higher in the skin of both the standard and FA hydrogel-treated groups.

## Discussion

Ferulic acid (FA) is a hydroxycinnamic acid phytoconstituent is richly found in fruits, vegetables, and cereals. FA has intrinsic biological activity including antioxidant, anti-inflammatory, collagen-promoting, angiogenic, and re-epithelialization effects in wound healing^49^. It falls within the classification of biopharmaceutical classification system (BCS) class II^9^. The oral bioavailability of FA is low, and research indicates that FA coupled to indigestible polysaccharides exhibits a poor intrinsic distribution rate ^50^. Free FA encounters numerous obstacles when delivered in conventional dosage forms due to its short biological half-life, the necessity for frequent dosing, breakdown in the stomach, first-pass metabolism, and inadequate permeability across biological membranes. Consequently, numerous scientists have developed advanced drug delivery techniques to surmount these obstacles ^11,51^. Studies on FA topical formulations indicate that formulation technologies enhance absorption and stability, hence improving therapeutic efficacy without supplanting the compound’s inherent action ^52^. Prior research has shown that FA-loaded nanocarriers exhibit improved stability, regulated release, and increased permeability relative to traditional formulations ^25^. The literature on nanoformulations for psoriasis and topical distribution indicates that increased efficacy frequently arises from enhanced skin penetration and retention through the stratum corneum barrier. ^53^.

In this study a FA-NE hydrogel was formulated with the aim of improving solubility and permeability for the topical treatment of psoriasis in a rat model. The selected FA concentration aligned with previously published levels for topical antioxidant and anti-inflammatory effects^13^. The excipients chosen for the study included castor oil, Smix (tween-80, propylene glycol). The number of NE formulations were prepared with varying proportions of Smix (1:1, 1:2, and 2:1) by using a pseudo ternary phase diagram approach, with appropriate ratios of oil phase to get stable NE formulations. The maximum NE region was found with oil & Smix ratios (5:5 and 6:4) which were used for the drug loaded NE formulation (NE1 to NE6). The NE regions were observed for transparency and uniformity and techniques such as conductivity birefringence, or systematic droplet size mapping prior to drug loading were not performed in this study. In literature numerous formulation studies have selected NE regions predominantly based on visual clarity and stability during titration analysis ^15,54,55^. An earlier study involved the preparation of a FA-loaded NE-based hydrogel, where the NE was evaluated based on its transparency and flowability. We adopted the same methodology, and after constructing phase diagrams using the aqueous titration method, we identified the NE phase based on transparency and uniformity^14^ The optimized NE5 was selected to formulate hydrogel, based on small droplet size, suitable zeta potential, and polydispersity index (Table 6). Direct application of NE on skin is generally not suitable due to its low viscosity and high flowability, which reduces the contact time with skin. Incorporating the NE into hydrogel matrix enhances its viscosity, consistency and prolongs the residence time at the application site^56^. A prior study indicated that FA NE incorporated into gels exhibited enhanced stability relative to the NE alone, increased the penetration of FA into the epidermis, prolonged skin residence time, and diminished irritative potential^57^. Carbopol-940 was utilized as the gelling agent to improve skin permeability. There were five different formulations (HG1-HG5) produced and HG5 was selected for further animal studies based on favorable rheology, permeation study findings (Fig. 2), and pH (Table 7). The rheological investigations indicated that the viscosity of HG5 was higher relative to the other hydrogels, likely due to the effective integration of the drug into the gel matrix, which led to an increase in gel viscosity. The rheogram of HG5 (Fig. 2) exhibited shear-thinning behavior, indicating that the viscosity of the nanoemulgel diminishes with an increase in shear rate. This behavior results in a thick formulation when maintained in the container, although it readily thins upon application to the skin, facilitating ease of use. HG5 demonstrated thixotropy, characterized by time-dependent recovery, wherein its structure disintegrates under shear but restores over time upon the removal of shear force. Thixotropy guarantees that the formulation is readily dispensable, distributes effectively, and rapidly restores its structure, thereby preventing the formulation from dripping off the skin. Thixotropic behavior is caused by the arrangement of asymmetric molecules within a material as it flows. The system (HG5) exhibit diminished apparent viscosity upon the application of shear force ^23^. The structural attributes of the FA-NE loaded hydrogel were assessed via SEM and FTIR following gel incorporation, yielding corroborative evidence of the uniform distribution and maintenance of the dispersed system within the hydrogel matrix. SEM analysis revealed a uniform and continuous surface morphology, devoid of any apparent phase separation, aggregation, or structural collapse, thereby indicating that the integration into the carbopol hydrogel did not significantly destabilize the NE system (Fig. 4). The particle size analysis post-hydrogel incorporation was not conducted in this study. A prior study demonstrated that the conversion of NEs into nanoemulgels resulted in only minor increase in particle size post-gel incorporation, with majority of formulations retaining droplet sizes under 100 nm, thereby indicating the preservation of nanostructural integrity within the gel network ^58^. In a similar manner, recent studies on carbopol-based nanoemulgels indicate that polymeric gel matrices can impede droplet diffusion without causing substantial instability or droplet coalescence. Research on carbopol 934/940 nanoemulgels indicates that the gel network primarily enhances viscosity, spreadability, and topical retention, while ensuring satisfactory physical stability and uniformity of the dispersed droplets ^59^.

In the in-vitro skin permeability study, HG5 showed significantly enhanced penetration through the skin, likely due to its ability to promote rapid water diffusion and enable efficient drug release from the formulation across the skin barrier. Likewise, the selected oil and Smix ratio used in the gel formulation may reduce the skin’s diffusion barrier and promote moisture retention, thereby enhancing drug penetration through the skin layer. Although specific membrane integrity testing using marker permeation or electrical resistance measurements was not performed, membranes were visually inspected and equilibrated before experimentation. The experimental design followed established Franz diffusion methodologies without explicit integrity testing or quantitative sink-condition verification reported in the literature^14,25^. Moreover, dispersion stability tests were conducted on all NE formulations and selected hydrogel at specified time intervals and were found stable. The stability analysis undertaken in this research was primarily to assess the physical stability and formulation integrity of the proposed nanoformulation during storage, rather than to determine a final commercial shelf-life profile. The formulated preparation was intended for short-term application within 14 days post-preparation; thus, the 6-month stability evaluation was conducted primarily to detect possible physical instability issues, including phase separation, precipitation, alterations in transparency, color changes, and pH fluctuations. In colloidal and NE-based systems, visual appearance and pH are widely recognized as sensitive indicators of physical destabilization, as changes in these parameters often reflect droplet aggregation, coalescence, or polymeric matrix disruption. In a previous study FA-NE hydrogel had shown sustained release of FA and improved drug permeability against ultraviolet radiation in an animal model. The stability studies of FA-NE3 hydrogel were not performed in this published research and only NEs was tested for its stability^14^, however in current study we evaluated short-term formulation stability and physical robustness of FA-NE hydrogel. We were unable to conduct drug content analysis and rheological characterization during storage, therefore we suggest to perform the analysis in future to perform the translational studies on the formulated FA-NE hydrogel ^14^. A subsequent research integrating FA into hydrogels, particularly dextran-based systems, has shown enhanced stability and transdermal delivery of additional antioxidants, including vitamin E ^60^.

Psoriasis is a common autoimmune disease caused by T-cell-mediated skin inflammation. In addition to significant inflammatory changes, psoriasis alters epidermal growth and differentiation. Topical, oral, systemic, and light treatments for psoriasis are available. Psoriasis treatment has been studied using several phytochemicals and herbal substances. Herbal medications have less adverse effects, lower costs, and more modes of action than conventional therapy ^61^. The FA-NE hydrogel HG5 was assessed in a psoriasis rat model, applied topically to the shaved dorsal region for 14 days, followed by a 7-day induction period using IMQ cream. IMQ, a strong immune stimulant, is applied topically for the treatment of vaginal and perianal warts caused by human papillomavirus infection. The activation of monocytes, macrophages, and plasmacytoid dendritic cells results in an increased production of proinflammatory cytokines and chemokines. This process subsequently enhances the recruitment of immune cells to the application site, leading to the proliferation of keratinocytes ^62^. In the current study the dosage of FA (10 mg/kg) was chosen in accordance with previously documented results ^4^. It was seen that the application of IMQ resulted in a reduction in antioxidant levels, specifically those of SOD, CAT, and GSH, alongside an elevation in inflammatory and oxidative biomarkers, namely IL-17, IL-23, and MDA, within both the skin and serum. The findings of the study indicated that the application of IMQ elicited an inflammatory response akin to the characteristics of psoriasis and oxidative stress condition. The application of IMQ via the IL-17 axis induces psoriasis-like lesions characterized by scaling and skin thickening, closely mirroring the pathophysiology seen in human psoriasis ^4^. In the treatment group, the levels of inflammatory biomarkers associated with oxidative stress were significantly reduced, like what was observed in the normal and standard groups. The observed favorable effects may be closely associated with the antioxidant and anti-inflammatory characteristics of FA, as documented in previous publications ^14^. They control inflammation, which leads to variable degrees of psoriasis growth and endothelial cell damage. The important effector cytokines are IL-23, IL-17 and TNF-α. TNF-α is believed to have a substantial role in supporting the cellular production of cytokines and enhancing their effects. IL-17 A exerts its primary effects on keratinocytes, leading to two significant outcomes. Firstly, it stimulates the synthesis of numerous inflammatory mediators. Secondly, it facilitates the aberrant proliferation of keratinocytes, hence causing epidermal hyperplasia, a characteristic manifestation of psoriasis ^63^. Prior studies have demonstrated that the interleukin IL-23/IL-17 pathway plays a significant role in the pathogenesis of psoriasis. The production of TNF-α, IL-17, IL-22, and various other cytokines is induced in Th17 cells by the release of IL-23 and IL-12 from inflammatory myeloid dendritic cells. The cytokines exert a significant impact on keratinocytes, hence intensifying the inflammatory response observed in psoriasis. The involvement of IL-23/Th17 in psoriasis is most convincingly demonstrated through clinical investigations. In psoriasis, interleukin IL-17 A, either independently or synergistically with TNF-α, can stimulate gene expression in keratinocytes, resulting in the activation of transcription factors such as STAT1 (signal transducer and activator of transcription 1) and nuclear factor-κB (NF-κB) ^64^. Based on the molecular docking results across key pro-inflammatory targets such as IL-6, IL-23, IL-17 A, TNF-α, and Ki-67, FA demonstrated significant anti-inflammatory potential. FA exhibited strong binding affinities and favorable interaction profiles within the TNF-α active site with docking scores of -6.6 kcal/mol. Furthermore, the stable conventional hydrogen bonding as well as π–π-hydrogen bonding interactions with critical amino acid residues indicate its potential to effectively disrupt TNF-α signaling. By forming hydrogen bond with TYR227 and SER136 (Fig. 5 7JRA-FA), FA prevents the binding of TNF-α to its receptor, thereby inhibiting the nuclear factor kappa-B pathway which is triggered by TNF-α. IL-17 A is a homodimer pro-inflammatory cytokine whose biological activity depends on the correct alignment of two subunits to bind to the IL-17 A receptor. The docking results showed ionic interaction of the carboxylate residue of FA with basic amino acid residue whereas lacking interaction with key amino acid residues is the core of pharmacological efficacy. The inhibition of formation of stabilized bonds by the FA causes steric hinderance, therefore IL-17 A can no longer bind to its receptor. The masking of p19 subunit by FA with IL-23 (3DHU) causes hinderance of IL17/Th17 chronic autoimmune axis ^4,5^. It can be suggested that phenolic ring of FA occupies the hydrophobic pockets and reinforces that position with its carboxylate group thereby disrupting the structural integrity of the cytokines to recognize and bind to their relevant receptors. This effectively silences the inflammatory signals, and these findings are consistent with the in-vivo observations of the cytokines (Fig. 8), suggesting the anti-inflammatory activity of FA-NE hydrogel in psoriasis model. The histological analysis demonstrated that the application of IMQ cream on the dorsal skin of rats resulted in an increase in thickness and hyperkeratosis in the shaved area of the diseased group. The histological characteristics of psoriatic lesions include several essential features like an increased epidermal thickness, a reduced or absent granular layer, known as hypogranulosis, due to abnormal keratinocyte differentiation, significant dilation of blood vessels in the papillary dermis, causing erythema, an elevated presence of neutrophils in the stratum corneum and a substantial accumulation of inflammatory cytokines in both the dermis and epidermis ^40^. Conversely, the treatment group exhibited a smooth skin surface without any visible lesions. The application of FA-NE hydrogel resulted in considerable improvement in the psoriasis area severity index. The expression levels of anti-Ki-67 were investigated as anti-Ki-67 plays a crucial function in cell proliferation. The expression of these proteins in the psoriatic skin lesion is significantly higher in comparison to the treatment, normal, and standard groups (Fig. 10). FA-NE hydrogel exhibited anti-psoriatic properties due to improved permeability and comparable results to the conventional immunosuppressant treatment available. Our study had specific constraints. The NE was identified using conventional methods of size distribution and zeta potential and we were unable to perform the experiments for in depth exploration of NE properties. Our studies also lack pharmacokinetic evaluation of the dose adjusted however various biochemical evaluations were conducted to confirm the therapeutic response. were unable to confirm particle shape and uniformity using TEM or cryo-SEM due to the unavailability of these facilities. The absence of additional techniques such as conductivity analysis, birefringence studies, or systematic pre-loading droplet size mapping, in-vitro skin integrity testing represents a limitation of the current work. Future investigations may incorporate these complementary analytical methods to further strengthen microstructural characterization of the NE system. Additionally, the work was limited to laboratory-scale preparation and short-term stability testing and was conducted only in an animal model. Long-term safety, human skin permeability, pharmacokinetic evaluation and dose–response effects remain to be explored in future studies.

## Conclusion

Ferulic acid nanoemulsion was successfully formulated and optimized for particle size, polydispersity index, zeta potential, entrapment efficiency, pH, and stability, then incorporated into a hydrogel and evaluated for skin permeation, pH, spreadability, and stability. The optimized FA-NE hydrogel significantly reduced inflammation, oxidative stress, and histopathological alterations in an imiquimod-induced psoriasis rat model. It lowered pro-inflammatory cytokines, enhanced antioxidant markers, decreased PASI scores, reduced keratinocyte desquamation, and downregulated Ki-67 expression, while also normalizing spleen index values. This study demonstrates strong therapeutic potential for FA-NE hydrogel as a topical psoriasis treatment.

## Supplementary Information

Below is the link to the electronic supplementary material.

Supplementary Material 1

## Data Availability

All data supporting the findings of this study are available within the paper and its Supplementary Information.

## References

[1] Bhat, M., Pukale, S., Singh, S., Mittal, A. & Chitkara, D. Nano-enabled topical delivery of anti-psoriatic small molecules. *J. Drug Deliv. Sci. Technol.* **62**, 102328. 10.1016/j.jddst.2021.102328 (2021).

[2] Dhabale, A. & Nagpure, S. Types of psoriasis and their effects on the immune system. *Cureus* **14**10.7759/cureus.29536 (2022). 10.7759/cureus.29536 PMC9592057 36312680

[3] Liu, C. et al. Kaempferol attenuates imiquimod-induced psoriatic skin inflammation in a mouse model. *Clin. Experimental Immunol.* **198**, 403–415. 10.1111/cei.13363 (2019). 10.1111/cei.13363 PMC6857081 31407330

[4] Lo, H. Y. et al. Ferulic acid altered IL-17A/IL-17RA interaction and protected against imiquimod-induced psoriasis-like skin injury in mice. *Food Chem. Toxicol.* **129**, 365–375. 10.1016/j.fct.2019.04.060 (2019). 31054998 10.1016/j.fct.2019.04.060

[5] Yang, J., Zhu, J., Lu, S., Qin, H. & Zhou, W. Transdermal psoriasis treatment inspired by tumor microenvironment-mediated immunomodulation and advanced by exosomal engineering. *J. Controlled Release*. **382**, 113664. 10.1016/j.jconrel.2025.113664 (2025). 10.1016/j.jconrel.2025.113664 40147535

[6] Dabholkar, N., Rapalli, V. K. & Singhvi, G. Potential herbal constituents for psoriasis treatment as protective and effective therapy. *Phytother. Res.* **35**, 2429–2444 (2021). 33277958 10.1002/ptr.6973

[7] Kaur, R. & Ajitha, M. Formulation of transdermal nanoemulsion gel drug delivery system of lovastatin and its in vivo characterization in glucocorticoid induced osteoporosis rat model. *J. Drug Delivery Sci.* **52**, 968–978 (2019).

[8] Baveloni, F. G. et al. Nanotechnology-based drug delivery systems as potential for skin application: a review. *Curr. Med. Chem.* **28**, 3216–3248. 10.2174/0929867327666200831125656 (2021). 32867631 10.2174/0929867327666200831125656

[9] Truzzi, F., Tibaldi, C., Zhang, Y., Dinelli, G. & D′ Amen, E. An overview on dietary polyphenols and their biopharmaceutical classification system (BCS). *Int. J. Mol. Sci.* **22**, 5514. 10.3390/ijms22115514 (2021). 34073709 10.3390/ijms22115514 PMC8197262

[10] Babbar, R., Dhiman, S., Grover, R., Kaur, A. & Arora, S. A comprehensive review on therapeutic applications of ferulic acid and its novel analogues: A brief literature. *Mini Rev. Med. Chem.* **21**, 1578–1593 (2021). 33494676 10.2174/1389557521666210120111702

[11] Purushothaman, J. R. & Rizwanullah, M. & Purushothaman Sr, R. Ferulic acid: a comprehensive review. *Cureus* **16** (2024). 10.7759/cureus.68063 PMC11438535 39347187

[12] Shukla, D. et al. Enhanced antichemobrain activity of amino acid assisted ferulic acid solid dispersion in adult zebrafish (Danio rerio). **14**, 3422–3437 (2024). 10.1007/s13346-024-01546-5 38573496

[13] Cavalcanti, G. R., Duarte, F. I. & Converti, A. Lima, Á. A. Ferulic acid activity in topical formulations: technological and scientific prospecting. *Curr. Pharm. Design*. **27**, 2289–2298. 10.2174/1381612826666201020163331 (2021). de. 10.2174/1381612826666201020163331 33081675

[14] Harwansh, R. K., Mukherjee, P. K., Bahadur, S. & Biswas, R. Enhanced permeability of ferulic acid loaded nanoemulsion based gel through skin against UVA mediated oxidative stress. *Life Sci.* **141**, 202–211. 10.1016/j.lfs.2015.10.001 (2015). 26437269 10.1016/j.lfs.2015.10.001

[15] Wang, X., Anton, H., Vandamme, T. & Anton, N. Updated insight into the characterization of nano-emulsions. *Expert Opin. Drug Deliv.* **20**, 93–114. 10.1080/17425247.2023.2154075 (2023). 36453201 10.1080/17425247.2023.2154075

[16] Gul, U. et al. Olive oil and clove oil-based nanoemulsion for topical delivery of terbinafine hydrochloride: in vitro and ex vivo evaluation. *Drug Deliv.* **29**, 600–612 (2022). 35174738 10.1080/10717544.2022.2039805 PMC8856056

[17] Zhao, S. et al. Characterization of nanoemulsions stabilized with different emulsifiers and their encapsulation efficiency for oregano essential oil: Tween 80, soybean protein isolate, tea saponin, and soy lecithin. *Foods* **12**, 3183 (2023). 37685117 10.3390/foods12173183 PMC10487023

[18] Oliveira, A. E. et al. Utilization of dynamic light scattering to evaluate Pterodon emarginatus oleoresin-based nanoemulsion formation by non-heating and solvent-free method. *Revista Brasileira de Farmacognosia*. **27**, 401–406. 10.1016/j.bjp.2016.11.005 (2017).

[19] Harwansh, R. K., Mukherjee, P. K., Bahadur, S. & Biswas, R. Enhanced permeability of ferulic acid loaded nanoemulsion based gel through skin against UVA mediated oxidative stress. *Life Sci.* **141**, 202–211 (2015). 26437269 10.1016/j.lfs.2015.10.001

[20] Mushtaq, A. et al. Development and Characterization of Heparin–Pullulan Liposomal Nano-Gel for Enhanced Silymarin Delivery in Dementia Therapy: In Vivo Evaluation in Albino Mice. *Pharmaceutics* **18**, 348. 10.3390/pharmaceutics18030348 (2026). 41900833 10.3390/pharmaceutics18030348 PMC13028882

[21] Elsewedy, H. S., Shehata, T. M. & Soliman, W. E. Tea tree oil nanoemulsion-based hydrogel vehicle for enhancing topical delivery of neomycin. *Life* **12**, 1011 (2022). 35888099 10.3390/life12071011 PMC9317510

[22] Laxmi, M., Bhardwaj, A., Mehta, S. & Mehta, A. Development and characterization of nanoemulsion as carrier for the enhancement of bioavailability of artemether. *Artif. cells Nanomed. Biotechnol.* **43**, 334–344. 10.3109/21691401.2014.887018 (2015). 24641773 10.3109/21691401.2014.887018

[23] Sultana, M. et al. Effect of Rheology and Poloxamers Properties on Release of Drugs from Silicon Dioxide Gel-Filled Hard Gelatin Capsules—A Further Enhancement of Viability of Liquid Semisolid Matrix Technology. *AAPS PharmSciTech*. **18**, 1998–2010. 10.1208/s12249-016-0674-0 (2017). 27933585 10.1208/s12249-016-0674-0

[24] Sevinç-Özakar, R., Seyret, E., Özakar, E. & Adıgüzel, M. C. Nanoemulsion-based hydrogels and organogels containing propolis and dexpanthenol: Preparation, characterization, and comparative evaluation of stability, antimicrobial, and cytotoxic properties. *Gels* **8**, 578 (2022). 36135290 10.3390/gels8090578 PMC9498717

[25] Das, S. & B Wong, A. Stabilization of ferulic acid in topical gel formulation via nanoencapsulation and pH optimization. *Sci. Rep.* **10**, 12288. 10.1038/s41598-020-68732-6 (2020). 32703966 10.1038/s41598-020-68732-6 PMC7378829

[26] Abioye, A. O., Issah, S. & Kola-Mustapha, A. T. Ex vivo skin permeation and retention studies on chitosan–ibuprofen–gellan ternary nanogel prepared by in situ ionic gelation technique—a tool for controlled transdermal delivery of ibuprofen. *Int. J. Pharm.* **490**, 112–130. 10.1016/j.ijpharm.2015.05.030 (2015). 25997660 10.1016/j.ijpharm.2015.05.030

[27] Arora, R., Aggarwal, G., Harikumar, S. & Kaur, K. Nanoemulsion based hydrogel for enhanced transdermal delivery of ketoprofen. *Advances in Pharmaceutics* 468456, (2014). 10.1155/2014/468456 (2014).

[28] Kasparaviciene, G. et al. Development and Evaluation of Two-Phase Gel Formulations for Enhanced Delivery of Active Ingredients: Sodium Diclofenac and Camphor. *Pharmaceutics* **16**, 366. 10.3390/pharmaceutics16030366 (2024). 38543261 10.3390/pharmaceutics16030366 PMC10974502

[29] Wang, S. Q. et al. Design of dual inhibitors of human TNF-α and IL-6 with potentials for the treatment of rheumatoid arthritis. *Trop. J. Pharm. Res.* **18**, 2305–2312. 10.4314/tjpr.v18i11 (2019).

[30] Aldossari, R. M. et al. Insights on in-silico approaches for identifying potential bioactive inhibitors for TNF-α and IL-6 proteins associated with rheumatoid arthritis. *Arab. J. Chem.* **16**, 105200. 10.1016/j.arabjc.2023.105200 (2023).

[31] Patel, J. R., Joshi, H. V., Shah, U. A. & Patel, J. K. In silico screening of natural compounds to identify lead as interleukin 17A receptor blockers as antihypertensive agents. *Thai J. Pharm. Sci.* **44**, 168–173. 10.56808/3027-7922.2448 (2020).

[32] Gupta, A. et al. Cytokines inhibitory mechanism of Prunus domestica L.(Plum) peptides as potential immunomodulators against systemic lupus erythematosus: an in-silico screening. *Silico Pharmacol.* **12**, 12. 10.1007/s40203-023-00188-8 (2024). 10.1007/s40203-023-00188-8 PMC10866836 38370860

[33] Hassan, I., Zaman, A., Zafar, E. & Khan, M. Molecular Docking-Aided Identification of Natural Bioactive Molecules as Potential Cancer Cell Proliferation Inhibitors: Molecular Docking as Cancer Cell Inhibitors. *Futuristic Biotechnol.* 25–30. 10.54393/fbt.v4i02.105 (2024).

[34] Iqbal, A. et al. Modulatory effects of rutin and vitamin A on hyperglycemia induced glycation, oxidative stress and inflammation in high-fat-fructose diet animal model. *Plos one*. **19**, e0303060. 10.1371/journal.pone.0303060 (2024). 38723008 10.1371/journal.pone.0303060 PMC11081234

[35] Muntaha, S. et al. Biochemical, molecular characterization, antioxidant, and cytotoxicity of Dicliptera bupleuroides with potential drug discovery: in vitro and in silico analysis. *ChemistrySelect* **9**, e202304385. 10.1002/slct.202304385 (2024).

[36] Labhar, A. et al. Seasonal Variations in the Essential Oil Composition and Biological Activities of Wild Lavandula dentata L. *Nat. Prod. Commun.* **19**, 1934578X241230822. 10.1177/1934578X241230822 (2024).

[37] Azeem, M. et al. Design, synthesis, spectroscopic characterization, in-vitro antibacterial evaluation and in-silico analysis of polycaprolactone containing chitosan-quercetin microspheres. *J. Biomol. Struct. Dynamics*. **41**, 7084–7103. 10.1080/07391102.2022.2119602 (2023). 10.1080/07391102.2022.2119602 36069131

[38] Zhang, S. et al. Efficacy and safety of curcumin in psoriasis: preclinical and clinical evidence and possible mechanisms. *Front. Pharmacol.* **13**, 903160. 10.3389/fphar.2022.903160 (2022). 36120325 10.3389/fphar.2022.903160 PMC9477188

[39] Yang, Y. et al. An emerging role of proanthocyanidins on psoriasis: evidence from a psoriasis-like mouse model. *Oxidative medicine cellular longevity* 5800586 (2022). (2022). 10.1155/2022/5800586 PMC9200549 35720176

[40] Yang, Y. et al. An emerging role of proanthocyanidins on psoriasis: evidence from a psoriasis-like mouse model. *Oxidative Med. Cell. Longev.* **2022** (5800586). 10.1155/2022/5800586 (2022). 10.1155/2022/5800586 PMC9200549 35720176

[41] Jabeen, M. et al. Advanced characterization of imiquimod-induced psoriasis-like mouse model. *Pharmaceutics* **12**, 789 (2020). 32825447 10.3390/pharmaceutics12090789 PMC7558091

[42] Sun, L. et al. Enhanced topical penetration, system exposure and anti-psoriasis activity of two particle-sized, curcumin-loaded PLGA nanoparticles in hydrogel. *J. Controlled Release*. **254**, 44–54 (2017). 10.1016/j.jconrel.2017.03.385 28344018

[43] Jabeen, M. et al. Advanced characterization of imiquimod-induced psoriasis-like mouse model. **12**, 789 (2020). 10.3390/pharmaceutics12090789 PMC7558091 32825447

[44] Shahbaz, M., Kamran, S. H. & Anwar, R. Amelioration of bleomycin and methotrexate-induced pulmonary toxicity by serratiopeptidase and fisetin. *Nutr. Cancer*. **73**, 2774–2784. 10.1080/01635581.2020.1860242 (2021). 33353415 10.1080/01635581.2020.1860242

[45] Kim, S. et al. Differential expression of cyclin D1, Ki–67, pRb, and p53 in psoriatic skin lesions and normal skin. *Mol. Med. Rep.* **17**, 735–742. 10.3892/mmr.2017.8015 (2018). 29115643 10.3892/mmr.2017.8015 PMC5780150

[46] Rowe, R. C. *Handbook of pharmaceutical excipients* (Pharmaceutical, 2020).

[47] Fiume, M. M. et al. Safety assessment of propylene glycol, tripropylene glycol, and PPGs as used in cosmetics. *Int. J. Toxicol.* **31**10.1177/1091581812461381 (2012). 245S-260S, doi. 10.1177/1091581812461381 23064775

[48] Tai, Q. D. et al. Glucose-responsive nanozyme hydrogel for glycemic control and catalytic anti-infective therapy in diabetic wound healing. *Mater. Today Bio*. **102405**10.1016/j.mtbio.2025.102405 (2025). 10.1016/j.mtbio.2025.102405 PMC12550175 41142415

[49] Cerqueira, A. F. L. W., de Mello Brandão, H., Tavares, G. D. & Rodarte, M. P. Ferulic Acid: A Review of Mechanisms of Action, Absorption, Toxicology, Application on Wound Healing. *Anti-Inflammatory & Anti-Allergy Agents in Medicinal Chemistry* 23, 205–214, (2024). 10.2174/0118715230309592240723105514 10.2174/0118715230309592240723105514 39108119

[50] Pyrzynska, K. Ferulic acid—a brief review of its extraction, bioavailability and biological activity. *Separations* **11**, 204. 10.3390/separations11070204 (2024).

[51] Li, D. et al. Ferulic acid: A review of its pharmacology, pharmacokinetics and derivatives. *Life Sci.* **284**, 119921. 10.1016/j.lfs.2021.119921 (2021). 34481866 10.1016/j.lfs.2021.119921

[52] Stompor-Gorący, M. & Machaczka, M. Recent advances in biological activity, new formulations and prodrugs of ferulic acid. *Int. J. Mol. Sci.* **22**, 12889 (2021). 34884693 10.3390/ijms222312889 PMC8657461

[53] Li, N. et al. Transdermal delivery of therapeutic compounds with nanotechnological approaches in psoriasis. *Front. Bioeng. Biotechnol.* **9**, 804415. 10.3389/fbioe.2021.804415 (2022). 35141215 10.3389/fbioe.2021.804415 PMC8819148

[54] Alhasso, B., Ghori, M. U. & Conway, B. R. Development of Nanoemulsions for Topical Application of Mupirocin. *Pharmaceutics* **15**, 378. 10.3390/pharmaceutics15020378 (2023). 36839700 10.3390/pharmaceutics15020378 PMC9960479

[55] Musakhanian, J. & Osborne, D. W. Understanding microemulsions and nanoemulsions in (trans) dermal delivery. *Aaps Pharmscitech*. **26**, 31. 10.1208/s12249-024-02997-2 (2025). 39794642 10.1208/s12249-024-02997-2

[56] Sah, A. et al. Design and Development of a Topical Nanogel Formulation Comprising of a Unani Medicinal Agent for the Management of Pain. *Gels* **9**, 794. 10.3390/gels9100794 (2023). 37888367 10.3390/gels9100794 PMC10606395

[57] de Almeida, T. M. et al. Ferulic acid-loaded Nanoemulsion Gels: A strategy for enhanced skin delivery and reduced irritation. *J. Drug Deliv. Sci. Technol.* **111**, 107199. 10.1016/j.jddst.2025.107199 (2025).

[58] Tošić, A. et al. Interfacial Molecular Interactions as Determinants of Nanostructural Preservation in Ibuprofen-Loaded Nanoemulsions and Nanoemulsion Gels. *Pharmaceutics* **17**, 1532. 10.3390/pharmaceutics17121532 (2025). 41471047 10.3390/pharmaceutics17121532 PMC12736200

[59] Zheng, Y. et al. Effects of Carbopol^®^ 934 proportion on nanoemulsion gel for topical and transdermal drug delivery: A skin permeation study. *Int. J. Nanomed.* 5971–5987. 10.2147/IJN.S119286 (2016). 10.2147/IJN.S119286 PMC5108606 27877042

[60] Cassano, R., Trombino, S., Muzzalupo, R., Tavano, L. & Picci, N. A novel dextran hydrogel linking trans-ferulic acid for the stabilization and transdermal delivery of vitamin E. *Eur. J. Pharm. Biopharm.* **72**, 232–238. 10.1016/j.ejpb.2008.10.003 (2009). 18976708 10.1016/j.ejpb.2008.10.003

[61] Di Caprio, R. et al. Safety concerns with current treatments for psoriasis in the elderly. *Exp. Opin. Drug Saf.* **19**, 523–531 (2020). 10.1080/14740338.2020.1728253 32056449

[62] Smajlović, A. et al. Molecular and histopathological profiling of imiquimod induced dermatosis in Swiss Wistar rats: contribution to the rat model for novel anti-psoriasis treatments. *Mol. Biol. Rep.* **48**, 4295–4303 (2021). 34097205 10.1007/s11033-021-06445-3

[63] Yamanaka, K., Yamamoto, O. & Honda, T. Pathophysiology of psoriasis: A review. *J. Dermatol.* **48**, 722–731 (2021). 33886133 10.1111/1346-8138.15913

[64] Nakajima, K. & Sano, S. Mouse models of psoriasis and their relevance. *J. Dermatol.* **45**, 252–263 (2018). 29226571 10.1111/1346-8138.14112

