# Supplementary material for: Preparation, characterization and in-vivo evaluation of nanoemulsion based hydrogel of ferulic acid in imiquimod-induced psoriasis in rats
Source: Sci Rep. 2026 Jun 10;16:26450. doi: 10.1038/s41598-026-57435-z (PMC13500538; doi:10.1038/s41598-026-57435-z)
Supplement: Supplementary file 1 [file 41598_2026_57435_MOESM1_ESM.docx]

**SUPPLEMEMTARY FILE**

**Preparation, Characterization and *In-Vivo* Evaluation of Nanoemulsion Based Hydrogel of Ferulic Acid in Imiquimod-Induced Psoriasis in Rats**

Nosheen Naseer^1^, Sairah Hafeez Kamran^1*^, Misbah Sultana^2^, Saiqa Ishtiaq^2^, Farhan Siddique^3^, Muhammad Farhan Sohail^4^, M. Ismail Mastoi^5^

^1^ Institute of Pharmacy, Faculty of Pharmaceutical and Allied Health Sciences, Lahore College for Women University, Lahore, 54000, Punjab, Pakistan

^2^ Punjab University College of Pharmacy, University of the Punjab, Lahore, 54000, Punjab, Pakistan

^3^ Department of Pharmaceutical Chemistry, Faculty of Pharmacy, Bahauddin Zakariya University 60800, Multan. Pakistan

^4^ Alliant College of Professional Studies, Lahore, Punjab, Pakistan

^5^ Allmed (Pvt.) Ltd. Sunder Industrial Estate, Raiwind Road, Lahore, Pakistan

**Corresponding Author**

*** Sairah Hafeez Kamran:** [**sairah.hafeez@lcwu.edu.pk**](mailto:sairah.hafeez@lcwu.edu.pk)

**ORCID: 0000-0002-3526-4132**

Table 1 Supp: Formulation Composition for Pseudo-Ternary Phase Diagram Development Using Smix (1:1)

| **Formulation** | **Oil & Smix** | **Oil** | | **Smix** | | **Smix (1:1)** | | | | **Water** | |
| --- | --- | --- | --- | --- | --- | --- | --- | --- | --- | --- | --- |
|  |  |  |  |  |  | **Surfactant** | | **Co-Surfactant** | |  |  |
|  |  | **%(V/V)** | **ml** | **%(V/V)** | **ml** | **%(V/V)** | **ml** | **%(V/V)** | **ml** | **%(V/V)** | **ml** |
| A1 | 1 : 9 | 4.0 | 0.8 | 36.0 | 7.2 | 18.0 | 3.6 | 18.0 | 3.6 | 60 | 12 |
| A2 | 2 : 8 | 9.0 | 1.8 | 36.0 | 7.2 | 18.0 | 3.6 | 18.0 | 3.6 | 55 | 11 |
| A3 | 3 : 7 | 16.5 | 3.3 | 38.5 | 7.7 | 19.3 | 3.85 | 19.3 | 3.85 | 45 | 9 |
| A4 | 4 : 6 | 22.0 | 4.4 | 33.0 | 6.6 | 16.5 | 3.3 | 16.5 | 3.3 | 45 | 9 |
| A5 | 5 : 5 | 22.5 | 4.5 | 22.5 | 4.5 | 11.3 | 2.25 | 11.3 | 2.25 | 55 | 11 |
| A6 | 6 : 4 | 30.6 | 6.1 | 20.4 | 4.1 | 10.2 | 2.04 | 10.2 | 2.04 | 49 | 9.8 |
| A7 | 7 : 3 | 34.3 | 6.9 | 14.7 | 2.9 | 7.4 | 1.47 | 7.4 | 1.47 | 51 | 10.2 |
| A8 | 8 : 2 | 38.4 | 7.7 | 9.6 | 1.9 | 4.8 | 0.96 | 4.8 | 0.96 | 52 | 10.4 |
| A9 | 9 : 1 | 45.0 | 9 | 5.0 | 1 | 2.5 | 0.5 | 2.5 | 0.5 | 50 | 10 |

Table 2 Supp: Formulation Composition for Pseudo-Ternary Phase Diagram Development Using Smix (1:2)

| **Formulation** | **Oil & Smix** | **Oil** | | **Smix** | | **Smix (1:2)** | | | | **Water** | |
| --- | --- | --- | --- | --- | --- | --- | --- | --- | --- | --- | --- |
|  |  |  |  |  |  | **Surfactant** | | **Co-Surfactant** | |  |  |
|  |  | **%(V/V)** | **ml** | **%(V/V)** | **ml** | **%(V/V)** | **ml** | **%(V/V)** | **ml** | **%(V/V)** | **ml** |
| A1 | 1 : 9 | 4.0 | 0.8 | 36.0 | 7.2 | 12.0 | 2.4 | 24.0 | 4.8 | 60 | 12 |
| A2 | 2 : 8 | 9.0 | 1.8 | 36.0 | 7.2 | 12.0 | 2.4 | 24.0 | 4.8 | 55 | 11 |
| A3 | 3 : 7 | 16.5 | 3.3 | 38.5 | 7.7 | 12.8 | 2.6 | 25.7 | 5.1 | 45 | 9 |
| A4 | 4 : 6 | 22.0 | 4.4 | 33.0 | 6.6 | 11.0 | 2.2 | 22.0 | 4.4 | 45 | 9 |
| A5 | 5 : 5 | 22.5 | 4.5 | 22.5 | 4.5 | 7.5 | 1.5 | 15.0 | 3.0 | 55 | 11 |
| A6 | 6 : 4 | 30.6 | 6.1 | 20.4 | 4.1 | 6.8 | 1.4 | 13.6 | 2.7 | 49 | 9.8 |
| A7 | 7 : 3 | 34.3 | 6.9 | 14.7 | 2.9 | 4.9 | 1.0 | 9.8 | 2.0 | 51 | 10.2 |
| A8 | 8 : 2 | 38.4 | 7.7 | 9.6 | 1.9 | 3.2 | 0.6 | 6.4 | 1.3 | 52 | 10.4 |
| A9 | 9 : 1 | 45.0 | 9 | 5.0 | 1 | 1.7 | 0.3 | 3.3 | 0.7 | 50 | 10 |

Table 2 Supp: Formulation Composition for Pseudo-Ternary Phase Diagram Development Using Smix (2:1)

| **Formulation** | **Oil & Smix** | **Oil** | | **Smix** | | **Smix (2:1)** | | | | **Water** | |
| --- | --- | --- | --- | --- | --- | --- | --- | --- | --- | --- | --- |
|  |  |  |  |  |  | **Surfactant** | | **Co-Surfactant** | |  |  |
|  |  | **%** | **%(V/V)** | **ml** | **%(V/V)** | **ml** | **%(V/V)** | **ml** | **%(V/V)** | **ml** | **%(V/V)** |
| A1 | 1 : 9 | 4.0 | 0.8 | 36.0 | 7.2 | 24.0 | 4.8 | 12.0 | 2.4 | 60 | 12 |
| A2 | 2 : 8 | 9.0 | 1.8 | 36.0 | 7.2 | 24.0 | 4.8 | 12.0 | 2.4 | 55 | 11 |
| A3 | 3 : 7 | 16.5 | 3.3 | 38.5 | 7.7 | 25.7 | 5.1 | 12.8 | 2.6 | 45 | 9 |
| A4 | 4 : 6 | 22.0 | 4.4 | 33.0 | 6.6 | 22.0 | 4.4 | 11.0 | 2.2 | 45 | 9 |
| A5 | 5 : 5 | 22.5 | 4.5 | 22.5 | 4.5 | 15.0 | 3.0 | 7.5 | 1.5 | 55 | 11 |
| A6 | 6 : 4 | 30.6 | 6.1 | 20.4 | 4.1 | 13.6 | 2.7 | 6.8 | 1.4 | 49 | 9.8 |
| A7 | 7 : 3 | 34.3 | 6.9 | 14.7 | 2.9 | 9.8 | 2.0 | 4.9 | 1.0 | 51 | 10.2 |
| A8 | 8 : 2 | 38.4 | 7.7 | 9.6 | 1.9 | 6.4 | 1.3 | 3.2 | 0.6 | 52 | 10.4 |
| A9 | 9 : 1 | 45.0 | 9 | 5.0 | 1 | 3.3 | 0.7 | 1.7 | 0.3 | 50 | 10 |

**Table 3 Supp: Determination of Ferulic Acid Solubility in Various Oil Phases (10 mL)**

| **Sr. No** | **Solubility in Castor Oil** | **Solubility Status** | **Solubility in Olive Oil** | **Solubility Status** | **Solubility in Isostearyl Isostearate ISIS** | **Solubility Status** |
| --- | --- | --- | --- | --- | --- | --- |
| 1. | 10mg | Freely Soluble | 10mg | Freely Soluble | 10mg | Freely Soluble |
| 2. | 10mg | Freely Soluble | 10mg | Freely Soluble | 10mg | Soluble |
| 3. | 10mg | Freely Soluble | 10mg | Soluble | 10mg | Saturated |
| 4. | 10mg | Soluble | 10mg | Saturated | Total qty. used 30mg | |
| 5. | 10mg | Saturated | Total qty. used 40mg | |  |  |
| 6. | Total qty. used 50mg | |  |  |  |  |

**Table 4 Supp: Determination of Ferulic Acid Solubility in Various Surfactant (10ml)**

| **Sr. No** | **Solubility in Tween-80** | **Solubility Status** | **Solubility in Labrasol** | **Solubility Status** |
| --- | --- | --- | --- | --- |
| 1. | 100mg | Highly Soluble | 100mg | Freely Soluble |
| 2. | 100mg | Freely Soluble | 100mg | Freely Soluble |
| 3. | 100mg | Freely Soluble | 100mg | Freely Soluble |
| 4. | 100mg | Freely Soluble | 100mg | Soluble |
| 5. | 100mg | Freely Soluble | 100mg | Saturated |
| 6. | 100mg | Freely Soluble | Total qty. used 500mg | |
| 7. | 100mg | Freely Soluble |  |  |
| 8. | 100mg | Soluble |  |  |
| 9. | Total qty. used 800mg | |  |  |

**Table 5 Supp: Determination of Ferulic Acid Solubility in Various Co-Surfactant (10ml)**

| **Sr. No** | **Solubility in Propylene Glycol** | **Solubility Status** | **Solubility in Glycerin** | **Solubility Status** |
| --- | --- | --- | --- | --- |
| 1. | 100mg | Freely Soluble | 100mg | Soluble |
| 2. | 100mg | Freely Soluble | 100mg | Saturated |
| 3. | 100mg | Freely Soluble | Total qty. used 200mg | |
| 4. | 100mg | Soluble |  |  |
| 5. | 100mg | Saturated |  |  |
| 6. | Total qty. used 500mg | |  |  |

**Fig 1 Supp: Schematic Representation of the Formulation Process**

**A**

**B**

**C**

**Fig. 2 Supp: Physicochemical Characterization of Nanoemulsion NE5 by Particle Size and PDI Analysis. A, B and C: three readings were taken of NE5**

Fig. 3 Supp: Dilution Test for Assessment of Nanoemulsion Stability

|  |  |  |  |  |
| --- | --- | --- | --- | --- |

Fig. 4 Supp: Visual assessment of Spreadibility of Gel formulations

**HPLC Analytical Method Validation Report of Ferulic Acid**

**HPLC Procedure:**

Estimation of FA content in the formulations was analyzed by RP-HPLC (Agilent Technologies) at a;

**Flow Rate** of 1 mL/min

**Run Time** 15 min at 321 nm wavelengths

**Temperature** 25 ± 0.5°C

**Column** Spherisorb C18 (250 mm × 4.6 mm, 5 μm; Waters, Ireland) fitted with a C18 guard column (10 × 3.0 mm).

**Injection Volume** 20μL

**Standard Stock Solution**

The standard stock solution was prepared by dissolving the known amount of FA in methanol to get a concentration of 5m g/mL. Different aliquots were prepared by subsequent dilution with the same solvent. Samples were stored at 4°C and protected from light before using.

**Mobile Phase**

In this analysis, mobile phase of acetonitrile and deionized water was used as a proportion of 35:65, 30:70 and 25:75 v/v (1% glacial acetic acid; pH 2.2) which were sonicated for 15 min and degassed prior to use.

Before applying all samples were also filtered through 0.45 μm (NYL) syringe filter. The method was validated as per the guideline of International Conference on Harmonization (ICH) Q2(R1) in terms of;

- Linearity & Range
- Accuracy
- Precision
- Specificity
- Limit of Detection
- Limit of Quantitation

The assay precision was calculated in terms of percentage of the relative standard deviation (% RSD) of determinations. Sensitivity of the system was determined by the limit of detection (LOD) and limit of quantification (LOQ) based on the standard deviation of the response (σ) and the slope (S) of the standard curve using the following equations.

LOD = 3.3σ/S and LOQ = 10σ/S.................. Eq. (1)

The recoveries of concentration pounds from various samples and formulation at three concentration levels were determined by measuring the percentages of detecting concentrations over additional concentrations. The quantitative analysis was repeated about three times (n = 3) by comparing and interpolating the peak area (response) of the sample with the standard.

1.1 **Linearity and Range:**

**Linearity:** Ability of the method to elicit test results that are directly proportional to the concentration of the analyte in samples within a given range.

**Range**: Lowest and the highest level of analyte that the method can determine with reasonable accuracy and precision.

**Determination of Linearity and Range**

The standard stock solution of Ferulic Acid was prepared in methanol to obtain a concentration of 5 mg/mL. Linearity was evaluated at five concentration levels (50–150%), corresponding to 2.50–7.50 mg/mL. The calibration curve was constructed by plotting peak area (AUC) versus concentration, which showed excellent linearity with a correlation coefficient (r²) of 0.9998.

| **S.#** | **AVG AUC of Standard for 5 injections = 605120** | | | | | **Correlation Coefficient (r^2^) and Y- intercept** |
| --- | --- | --- | --- | --- | --- | --- |
|  | **Conc. of** **Ferulic Acid** | | **Area under curve (AUC)** | **Conc. Recovered** | |  |
|  | **%age** | **mg/ml** |  | **%age** | **mg/ml** |  |
| 1. | 50 | 2.5 | 305000 | 50.4 | 2.52 | r^2^= 0.9998  Y=120000x + 5000 |
| 2. | 75 | 3.75 | 455200 | 75.2 | 3.76 |  |
| 3. | 100 | 5 | 605120 | 100.0 | 5.01 |  |
| 4. | 125 | 6.25 | 754980 | 124.8 | 6.24 |  |
| 5. | 150 | 7.5 | 905300 | 149.6 | 7.48 |  |

1.2 **Accuracy:**

“It relates to the closeness of the test results to the true value”

OR

“It is the measurement of the exactness of the analytical method.”

It is expressed as %age recovery by the known/added amount of the analyte in the linearity range.

Accuracy should be assessed using a minimum of nine determinations over a minimum of three concentration levels, covering the specified range. (i.e., three concentrations and three replicates of each concentration)

**Determination of Accuracy**

Prepare 50%, 75%, 100%, 125% & 150% of sample solution in triplicates, similarly prepare standard as per procedure and record your observations after analysis.

Acceptance Criteria: The relative standard deviation should not be more than 2.

Calculate the Standard Deviation & Relative Standard deviation by applying the following formula:

**Standard Deviation =	SD	=**	∑ (X_i_ − X)^2^

n−1

&

**Relative Standard Deviation** (Coefficient of Variation) =	100 × SD

X

| **S. No.** | **Avg. AUC of Standard for Five injections = 605120** | | | | | | **SD** | **RSD %**  **NMT 2** |
| --- | --- | --- | --- | --- | --- | --- | --- | --- |
|  | **Conc. of Ferulic Acid** | | **Area under Curve (AUC)** | **Recovery of Ferulic Acid (% age)** | **Ave. Recovery of Ferulic Acid** | |  |  |
|  | **% age** | **mg/ml** |  |  | **% age** | **mg/ml** |  |  |
| 1 | 50 | 2.5 | 305100 | 50.42 | 50.41 | 2.52 | 0.03 | 0.07 |
|  |  |  | 304800 | 50.37 |  |  |  |  |
|  |  |  | 305200 | 50.44 |  |  |  |  |
| 2 | 75 | 3.75 | 455300 | 75.24 | 75.24 | 3.76 | 0.03 | 0.03 |
|  |  |  | 455100 | 75.21 |  |  |  |  |
|  |  |  | 455400 | 75.26 |  |  |  |  |
| 3 | 100 | 5 | 605100 | 100.0 | 100.0 | 5.00 | 0.01 | 0.01 |
|  |  |  | 605200 | 100.01 |  |  |  |  |
|  |  |  | 605150 | 100.0 |  |  |  |  |
| 4 | 125 | 6.25 | 754800 | 124.74 | 124.78 | 6.24 | 0.04 | 0.03 |
|  |  |  | 755300 | 124.82 |  |  |  |  |
|  |  |  | 755100 | 124.79 |  |  |  |  |
| 5 | 150 | 7.5 | 906500 | 149.80 | 149.68 | 7.48 | 0.12 | 0.08 |
|  |  |  | 905600 | 149.66 |  |  |  |  |
|  |  |  | 905100 | 149.57 |  |  |  |  |

1.3 **Precision:**

“The precision of an analytical method is the degree of agreement among individual test results when the method is applied repeatedly to multiple samplings of a homogeneous sample.

OR

“It is the measure of the degree of repeatability or reproducibility under normal conditions. It is usually expressed as Standard Deviation (SD) or Relative Standard Deviation (RSD) or coefficient of variation of a series of measurements.

**Determination of Precision:**

Prepare sample of 100% concentration for five determinations from multiple samplings of a homogenous sample. Similarly prepare standard as per procedure and record your observations after analysis.

**Acceptance Criteria = The relative standard deviation should not be more than 2.**

Calculate the Standard Deviation & Relative Standard deviation by applying the following formula:

**Standard Deviation =	SD	=**	∑ (X_i_ − X)^2^

n−1

&

**Relative Standard Deviation** (Coefficient of Variation) =	100 × SD

X

| **Avg. AUC of Standard for Five injections: 605120** | | | | | | | | |
| --- | --- | --- | --- | --- | --- | --- | --- | --- |
| **Sr. No.** | **Conc. Of**  **Ferulic Acid** | | **Area Under Curve (AUC)** | **Conc. Recovered** | | **Typical Retention Time** | **SD** | **RSD %** |
|  | **%age** | **mg/ml** |  | **%age** | **mg/ml** |  |  |  |
| 1. | 100 | 5 | 605900 | 100.13 | 5.00 | 5.21 | 0.00 | 0.08 |
| 2. | 100 | 5 | 605200 | 100.01 | 2.49 | 5.22 |  |  |
| 3. | 100 | 5 | 605700 | 100.10 | 1.25 | 5.20 |  |  |
| 4. | 100 | 5 | 604480 | 99.89 | 0.63 | 5.21 |  |  |
| 5. | 100 | 5 | 605250 | 100.02 | 0.31 | 5.22 |  |  |
| 6. |  |  |  | 100.03 | 5.00 |  |  |  |
| 7. | Average Conc. Recovered: 5.00mg/ml | | | | Average % recovered: 100.03% | | | |

1.4 **Specificity:**

“It is the ability of the method to measure accurately and specifically the analyte of interest in the presence of matrix and other components likely to be present in the sample matrix and impurities, degradation products and other related substances.”

For this one may compare the test results of analysis of samples containing other ingredients/ impurities/ degradation products/ related substances/ placebo with those obtained from analysis of sample without these, i.e., the method allows distinct analytical measurement of analyte of interest and exclusion of all other relevant interferences.

**Determination of Specificity:**

Prepare sample & standard at 100% concentration; prepare also a sample without active (Placebo).

| **S. No.** | **Avg AUC of**  **100 % STD** | **AUC of**  **100 % SMP** | **% Recovery**  **of SMP** | **Percentage**  **Placebo** |
| --- | --- | --- | --- | --- |
| 1 | 605120 | 604980 | 99.98 | 0 |
| 2 |  | 605050 | 99.99 |  |

1.5 **Limit of Detection (LOD):**

“It is the lowest amount of the analyte in a sample that can be detected but not necessarily quantitated under the stated experimental conditions.

The limit of detection not only depends on the procedure of analysis but also on the type of instruments.

**Determination of Detection Limit:**

To determine the smallest quantity of analyte which can be detected by test procedure prepares five samples of different concentrations and compares the measured signals from samples with known low concentrations of analyte with those of blank samples. Establish the minimum concentration at which the analyte can reliably be detected.

**AVG AUC of Standard for Five injections. 605120**

| **S. No.** | **Conc. of Ferulic Acid** | | **AUC** | **Conc. Recovered** | | **Retention Time** |
| --- | --- | --- | --- | --- | --- | --- |
|  | **%age** | **mg/ml** |  | **%age** | **mg/ml** |  |
| 1. | 100 | 5 | 605400 | 100.05 | 5.00 | 5.21 |
| 2. | 50 | 2.50 | 301900 | 49.89 | 2.49 | 5.21 |
| 3. | 25 | 1.25 | 152000 | 25.12 | 1.26 | 5.22 |
| 4. | 12.5 | 0.63 | 76100 | 12.58 | 0.63 | 5.21 |
| 5. | 6.25 | 0.31 | 38020 | 6.28 | 0.31 | 5.22 |
| 6. | 3.125 | 0.16 | 18900 | 3.12 | 0.16 | 5.21 |

1.6 **Limit of Quantitation (LOQ):**

It is defined as the lowest amount of the analyte in a sample that can be determined with acceptable precision, accuracy and reliability under the stated experimental conditions. **Determination of Quantitation Limit:**

To determine the smallest quantity of analyte which can be quantified by test procedure, prepare five samples of different concentrations & standard at 100% concentration by following the recommended procedure and compare the measured signals from samples with known low concentrations of analyte with those of blank samples. Establish the minimum concentration at which the analyte can reliably be quantified.

**AVG AUC of Standard for 5 injections: 605120**

| **S. No.** | **Conc. of Ferulic Acid** | | **AUC** | **Conc. Recovered** | | **Remarks** |
| --- | --- | --- | --- | --- | --- | --- |
|  | **%age** | **mg/ml** |  | **%age** | **mg/ml** |  |
| 1. | 100 | 5 | 605120 | 100.0 | 5.00 | Ok |
| 2. | 50 | 2.50 | 304980 | 50.4 | 2.52 | Ok |
| 3. | 25 | 1.25 | 152500 | 25.2 | 1.26 | Ok |
| 4. | 12.5 | 0.63 | 75010 | 12.4 | 0.62 | Ok |
| 5. | 6.25 | 0.31 | 39020 | 6.4 | 0.32 | Ok |
| 6. | 3.125 | 0.16 | 20010 | 3.3 | 0.17 | Ok |

The limit of Quantitation (LOQ) of analyte (Ferulic Acid) is 3.125 % (0.16mg/ml) and recovered %age is 3.3 % (0.17mg/ml).
